# Insights into the palaeobiology of an early *Homo* infant: multidisciplinary investigation of the GAR IVE hemi-mandible, Melka Kunture, Ethiopia

**DOI:** 10.1038/s41598-021-02462-1

**Published:** 2021-11-29

**Authors:** Adeline Le Cabec, Thomas Colard, Damien Charabidze, Catherine Chaussain, Gabriele Di Carlo, Sabine Gaudzinski-Windheuser, Jean-Jacques Hublin, Rita T. Melis, Laura Pioli, Fernando Ramirez-Rozzi, Margherita Mussi

**Affiliations:** 1 Department of Human Evolution, Max Planck Institute for Evolutionary Anthropology, Deutscher Platz 6, 04103 Leipzig, Germany; 2 Univ. Bordeaux, CNRS, MCC, PACEA, UMR 5199, 33600 Pessac, France; 3 Department of Orthodontics, University of Lille, Lille University Hospital, 59000 Lille, France; 4 UMR 8025, Centre d’Histoire Judiciaire, University of Lille, 59000 Lille, France; 5 UR 2496 Orofacial Pathologies, Imaging and Biotherapies. Dental School Université de Paris, AP-HP- Hôpital Bretonneau - Service Odontologie CRMR Métabolisme du Phosphore et du Calcium (OSCAR, ERN Bond), Paris, France; 6 Department of Oral and Maxillofacial Sciences, Unit of Pediatric Dentistry, Sapienza University of Rome, Rome, Italy; 7 MONREPOS Archaeological Research Centre and Museum for Human Behavioural Evolution, Römisch-Germanisches Zentralmuseum, Leibniz-Forschungsinstitut Für Archäologie and Institute of Ancient Studies, Johannes Gutenberg–University Mainz, Schloss Monrepos, 56567 Neuwied, Germany; 8 Italian Archaeological Mission at Melka Kunture and Balchit, Rome, Italy; 9 Dipartimento Di Scienze Chimiche E Geologiche, Università Degli Studi Di Cagliari, Cittadella Universitaria, 09042 Monserrato (CA), Italy; 10 UMR 7206 CNRS MNHN UP Ecoanthropologie Musée de l’Homme, Paris, France; 11 Dipartimento di Scienze dell’Antichità, Università di Roma La Sapienza, Piazzale A. Moro 5, 00185 Rome, Italy

**Keywords:** Biological anthropology, Archaeology

## Abstract

Childhood is an ontogenetic stage unique to the modern human life history pattern. It enables the still dependent infants to achieve an extended rapid brain growth, slow somatic maturation, while benefitting from provisioning, transitional feeding, and protection from other group members. This tipping point in the evolution of human ontogeny likely emerged from early *Homo.* The GAR IVE hemi-mandible (1.8 Ma, Melka Kunture, Ethiopia) represents one of the rarely preserved early *Homo* infants (~ 3 years at death), recovered in a richly documented Oldowan archaeological context. Yet, based on the sole external inspection of its teeth, GAR IVE was diagnosed with a rare genetic disease–amelogenesis imperfecta (AI)–altering enamel. Since it may have impacted the child’s survival, this diagnosis deserves deeper examination. Here, we reassess and refute this diagnosis and all associated interpretations, using an unprecedented multidisciplinary approach combining an in-depth analysis of GAR IVE (synchrotron imaging) and associated fauna. Some of the traits previously considered as diagnostic of AI can be better explained by normal growth or taphonomy, which calls for caution when diagnosing pathologies on fossils. We compare GAR IVE’s dental development to other fossil hominins, and discuss the implications for the emergence of childhood in early *Homo*.

## Introduction

The pattern and timing of modern human life history is unique in involving an extended period of growth^1^. This provides more time not only for somatic development and protracted brain growth^2, 3^, but also to learn survival skills^4^. The offspring is thus dependent upon more parental care over a longer time, and sexual maturity is postponed^5^. In modern humans, life history stages successively involve infancy, childhood, a juvenile phase, adolescence, and finally adulthood. Different kinds of feeding strategies and dental development stages characterize these phases. Following these criteria, infancy can be subdivided into two stages. First, the “nursing phase” takes place from birth to 6 months, when the infant exclusively relies on breastfeeding and starts erupting its anterior deciduous teeth. Second, the “suckling phase” spans from 6 months to 3 years and involves supplementing complementary food to supply the nutrients not sufficiently present in quantity or quality in the mother milk to enable the fast postnatal brain growth, and the eruption of all deciduous teeth. Weaning is one of the pivotal life history traits occurring during ontogeny: this is a long process involving a transition from exclusive breastfeeding to the progressive introduction of solid food, towards total cessation of mother milk consumption (fully weaned infants) and full reliance on solid diet^6, 7^, and eventually feeding self-sufficiency. During hominin evolution, major changes in weaning and feeding behaviour may have occurred with early *Homo*, especially involving the reduction in dental size and dietary shifts to focus on food items more energy dense and easier to masticate, and on an increase in meat and fat consumption and food sharing^3, 8, 9^. Known as the “Expensive-Tissue Hypothesis”^8^, this energy-rich diet would have enabled meeting the requirements of this demanding postnatal brain growth and achieving adult brain size by the end of childhood in humans. Following infancy, “childhood” spreads from 3 to 6 years of age when modern human weaned infants show all of their deciduous teeth in functional occlusion, although they cannot yet fully process an adult diet, due to their relatively small size and thin enamel^5^. In modern humans, the end of childhood is marked by the eruption of the permanent first molar (M1) at ~ 6 years of age^1^, and the attainment of adult brain size^10^.

In this context, fossil teeth are especially valuable not only because their recovery enables comparing developmental stages between fossil hominins and modern humans but also because dental hard tissues record their own growth and maturation as well as stressful events experienced by the organism^11^. In the tooth microstructure, stress events may manifest as accentuated lines and remain non-specific^12–14^. A specific accentuated growth marking called the neonatal line is often identifiable in the enamel and dentine of the deciduous teeth and in the mesial cusps of the permanent first molar which start mineralizing in utero^15, 16^.

During both infancy and childhood, protection and food provisioning by adults of the group (parents or others) is crucial for the child’s survival. Understanding the modality and time of emergence of this unique modern life history pattern is currently one of the most debated topics in human evolution^9, 17, 18^.

**Figure 1:**
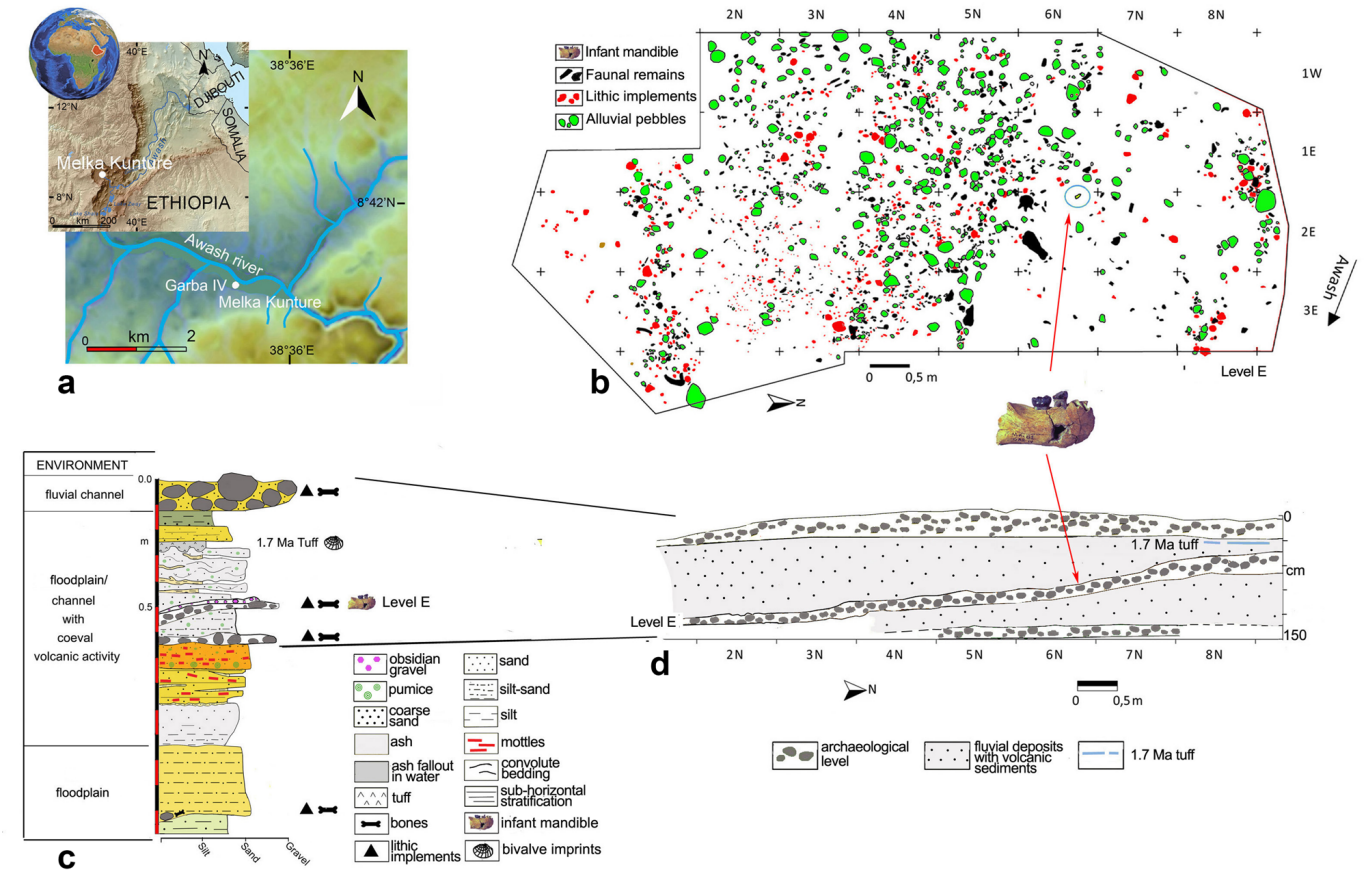
Garba IV site. (**a**) Location map of Melka Kunture and the Garba IV site (modified after https://upload.wikimedia.org/wikipedia/commons/2/24/Africa_satellite.jpg, https://commons.wikimedia.org/wiki/File:Awashrivermap.png, and https://data.humdata.org/dataset/ethiopia-elevation-model using QGIS software; https://qgis.org); (**b**) Plan of archaeological level E with position of GAR IVE (modified after Fig. 4A from Ref.^27^); (**c**) Stratigraphic log of Garba IV (Photoshop CS3; https://www.adobe-photoshop); (**d**) schematic cross-section of the archaeological levels (modified after Fig. 3B from Ref.^27^). The map of the Awash River (https://commons.wikimedia.org/wiki/File:Awashrivermap.png) is licensed under the Creative Commons Attribution-ShareAlike 3.0 license. The license terms can be found on the following link: http://creativecommons.org/licenses/by-sa/3.0/.

GAR IVE (full name: MK 81 GAR IVE 0043) is a fragmentary right hemimandible with a partial mixed dentition, recovered in layer E at the Garba IV site of the Melka Kunture complex (Upper Awash, Ethiopia) (Fig. 1). This fossil was attributed to *Homo erectus* s.l.^19^, and its age at death was estimated to be 2–3.5 years^20, 21^. With the recent discovery of *H. erectus* s.l. remains in association with Acheulean and Oldowan artifacts at Gona (Afar, Ethiopia)^22^, GAR IVE is one of the rare early *Homo* remains recovered from the beginning of the Early Pleistocene, in the context of proper archaeological excavations providing contextual information on hominin activities^23^. GAR IVE was found with Oldowan industry and faunal remains in layer E, later dated to ~1.8 Ma (Fig. 1b–d)^24–28^. The deposits of Garba IV were dated and arranged in sequence in a thoroughly established chronostratigraphy^26, 29^, which was evaluated again during fieldwork in 2013 and 2017.

Previous studies reported GAR IVE as being affected by a rare genetic disease *amelogenesis imperfecta* (AI)^19^, although they were only based on classical radiographs and SEM imaging of epoxy replicas of the tooth surface^19, 30^. Zanolli and colleagues worked on CT data, but did not re-evaluate this diagnosis^21^. Amelogenesis imperfecta encompasses a group of developmental conditions^31^ altering the enamel structure and making teeth fragile^32^. The main diagnostic criteria may involve a marked reduction in enamel thickness frequently concerning both deciduous and permanent teeth, enamel pits or linear hypoplasia not associated with any noticeable developmental defects in the other dental tissues, a brown colouration of the enamel, or taurodontism (enlargement of the pulp cavity associated with a lowering of the roof of the pulp chamber and root furcation level, giving the impression of a partial to complete fusion of the tooth roots)^31–36^. In some AI variants, enamel defects may be associated with other types of syndromes occurring in other parts of the human body (e.g., kidney diseases)^32, 37, 38^. In clinics, a differential diagnosis requires the knowledge of medical and family history of the patients, of their genetic profile, as well as of their conditions of development and life. Thus, AI patients mostly suffer from early tooth loss, severe embarrassment, eating difficulties and pain, requiring early and complex restorative treatments^38^.

Genetic diseases are of particular interest as they happen to be transmitted along a lineage affecting multiple generations, and they may occur with variable frequencies, in the case of AI, ranging from 1:700 to 1:14 000^38^. According to Zilberman et al.^19, 30^, the presence of AI in GAR IVE would provide evidence of genetic continuity from early *Homo* to modern humans. Yet, this claim has to be considered with caution since the molecular mechanisms causing this pathology are complex and may involve several genes and combinations of genes^39^. Such a genetic continuity is unlikely to be so straightforward. Furthermore, Trinkaus^40^ has suggested that any variant of this pathology would have involved the need for enhanced maternal care to supply the young child with sufficient nutrients and permit its survival to some extent^40^.

Given the putative consequences of this disease on the specimen’s morphology and the potential implications of living with AI during the Pleistocene, we aim to reassess the diagnosis of AI by analyzing tooth surface features and by exploring the fine internal structure of the bone and teeth using synchrotron imaging. We compare the GAR IVE teeth with teeth from modern human patients, some with documented forms of AI. Because taphonomic processes may affect the preservation of the specimens and the features related to the AI diagnosis, our study includes the analysis of the associated faunal remains and the geological context in which GAR IVE was recovered in order to establish if particular features are due to taphonomy or to developmental alterations induced by the AI. In spite of the taphonomic damages undergone by the dental tissues, we provide some novel fragmentary yet still valuable palaeobiological data on the dental development of this early *Homo* infant.

## Results

After a brief description of the GAR IVE specimen, and especially of its dental development, we have compared the enamel features described as diagnostic of AI on GAR IVE to the enamel condition in teeth of modern-day AI patients. Then, we have explored the fine microstructure of the teeth and bone in light of taphonomic processes, recovered from the study of associated faunal remains, and the palaeoenvironmental reconstruction of the fossilisation context of GAR IVE.

### Description of GAR IVE

**Figure 2:**
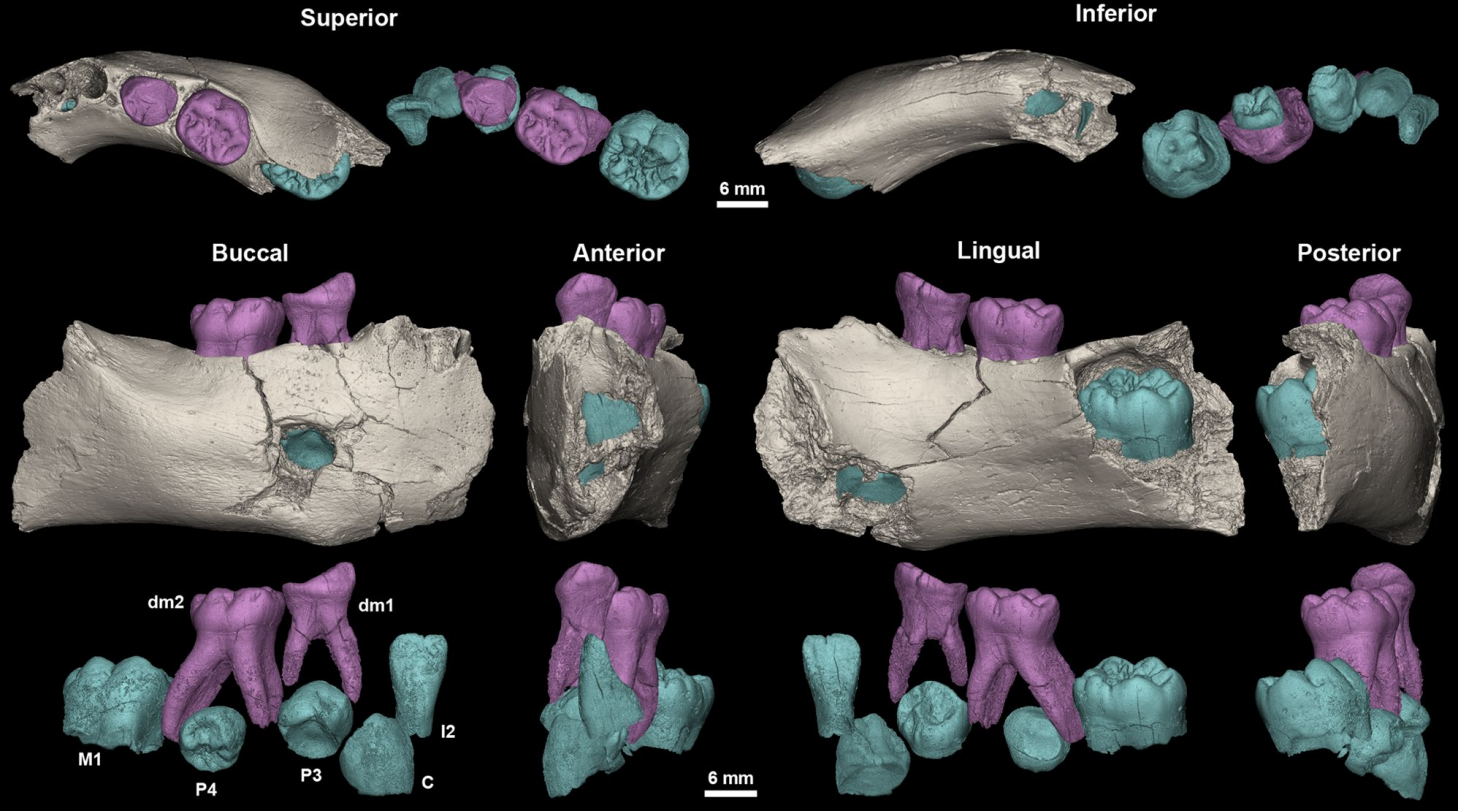
3D models of the GAR IVE right hemi-mandible showing the mandibular bone (beige), the preserved deciduous molars (dm1 and dm2; pink) and the germs of the permanent teeth (I2: lateral incisor, C: canine, P3 and P4: first and second premolar, M1: first molar; blue). Superior, inferior, buccal, anterior, lingual and posterior views.

**Figure 3:**
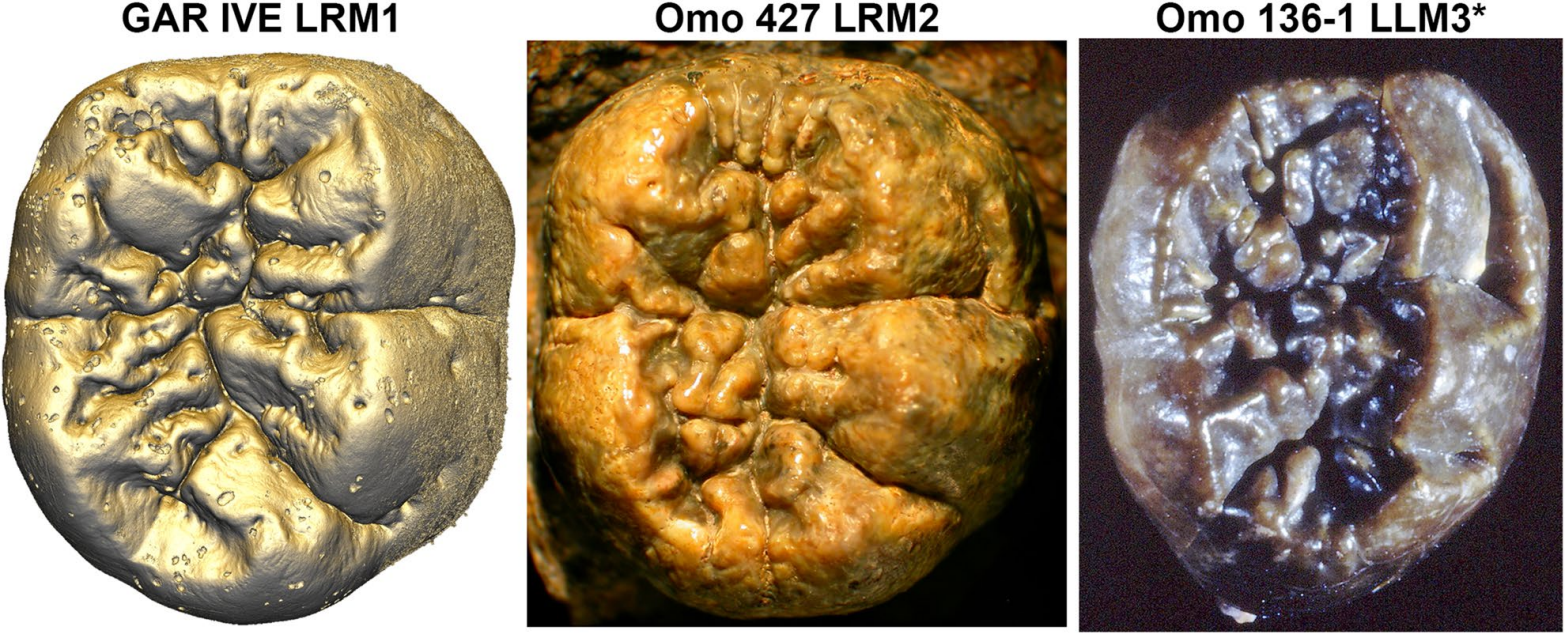
The occlusal surface of the LRM1 of GAR IVE (3D model) shows an amount of wrinkling comparable with that of other Plio-Pleistocene hominin molars such as the LRM2 of Omo 427 and the LLM3 of Omo 136-1 (*: flipped for illustrative purpose; both teeth were macro-photographed).

The GAR IVE right hemimandible (Fig. 2) preserves both erupted deciduous molars (LRdm_1_ and LRdm_2_). The LRdm_1_ is considerably worn (yet preserving a complete rim of enamel) and super-erupted above the occlusal level. The germ of the permanent first molar (LRM_1_) is partially exposed at the posterior part of the specimen, as the mandibular ramus is missing. Its crown does not present any abnormal morphological features; to note that its occlusal surface shows extensive wrinkling, yet this LRM_1_ looks similar to that of other fossil hominins (Fig. 3). The anterior portion of GAR IVE shows a greenstick fracture on its lingual side, suggesting this occurred peri-mortem, when the bone would have retained a certain level of collagen and moisture, thus still be fresh and elastic to some extent^41^. This fracture leaves the permanent lateral incisor (LRI_2_) visible and partially unprotected by its bony crypt. The germs of the permanent canine (LRC_1_), third and fourth premolars (LRP_3_ and LRP_4_) are unerupted and protected inside the mandibular bone. The bone and teeth present various kinds of surface alterations.

### Insights into GAR IVE’s dental development

**Figure 4:**
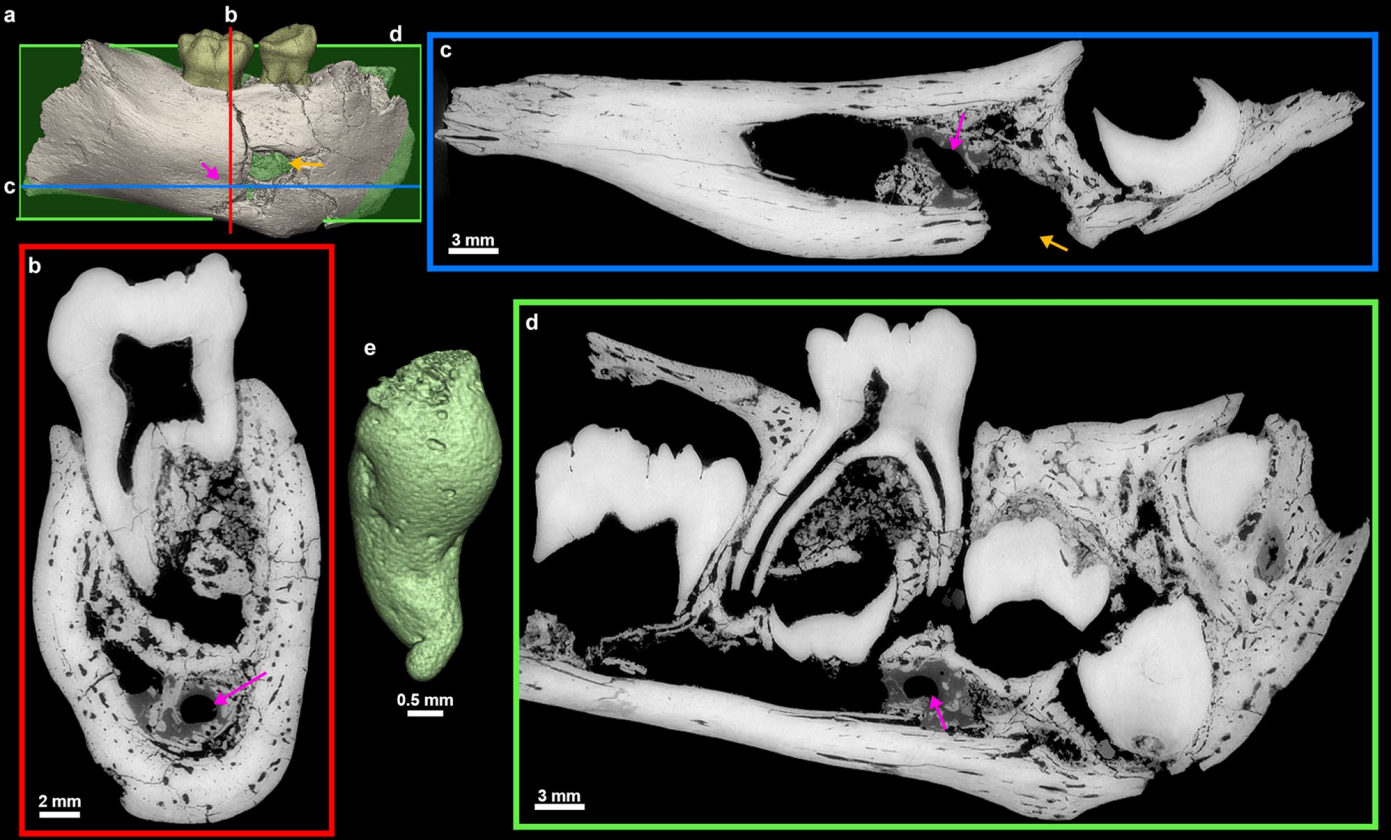
3D model of the GAR IVE specimen (**a**), and frontal (**b**, red), axial (**c**, blue) and sagittal (**d**, green) planes of section to illustrate the morphology of the hole (orange arrow) located in the area of the mental foramen. Its inner negative imprint shows a complex shape which is little compatible with the shape of a carnivore tooth (**c**). To note an additional convoluted shape represented in green in 3D (pink arrow).

The synchrotron µCT scans show a loss of contrast between bone, enamel and dentine, the enamel-dentine junction being barely visible in some places (Fig. 4). Any estimation of age at death based on dental growth increments and virtual histology techniques is thus precluded. At best, the maturation stage of the permanent tooth germs may provide insights into GAR IVE’s dental development (See Supplementary Text S1 for details). Following modern human standards^42^, the overall calcification stages in GAR IVE would correspond to 4.5 years of age (with individual scores per tooth going up to 7.5 years for the P_3_; see Supplementary Table S1). Using Kuykendall’s regression equation^43^, GAR IVE would be 3.13 [2.14–4.12] years in a chimpanzee-equivalent model. Several fossil hominins died at developmental stages similar to that of GAR IVE, although any comparison highlights some modularity in the relative advancement of each tooth type across taxa (See Supplementary Text S1). In terms of calcification stages, KNM-ER 812 (*Paranthropus boisei*) seems to be developmentally the closest to GAR IVE and has an estimated age at death of 2.5–3.0 years. KNM-ER 1820 (*P. boisei*) is slightly more advanced than GAR IVE for an age at death similar to KNM-ER 812 (2.5–3.1 years). Overall, the calcification of the GAR IVE permanent tooth germs is less advanced than that of Sts 24 (*Australopithecus africanus*).

In spite of the taphonomic alteration of the dental tissues in GAR IVE, the synchrotron data revealed several accentuated lines in the LRP4 (Supplementary Text S1, Supplementary Fig. S13). These accentuated markings may correspond to multiple stressful events. However, it remains impossible to ascertain their cause which may result from non-specific stress events in the life of the child. It also remains unknown whether these accentuated markings may witness disruptions in the growth process of GAR IVE, or even in its health. Assuming the initiation time of P4 (~ 2 years) and that the EDJ crest where the virtual slice was recorded formed ~ 3 months after the dentine horn tip, these were added to the times of formation of each stress line will yield an age of formation (Supplementary Text S1, Supplementary Table S9). Rough estimates following Dean et al.^44^ show that they occurred every ~ 1.4 months, from 2.5 to 2.9 years of age (see Supplementary Text S1 for details). When superimposed with the lingual cusp of the GAR IVE LRP4, the most developed cusp of the DNH 35 (South African *Homo*) LRP4 matches with the purple stress “S3” in GAR IVE (see Supplementary Text S1 and Supplementary Fig. S14), which, following the DNH 35 growth pattern^45^, would have then occurred at ~ the 796th day of life of GAR IVE (i.e., 2.18 years). Death occurred ~ 77 days later in GAR IVE, which correspond to ~ 873 days or ~ 2.4 years in the DNH 35 growth pattern. “S3” shows that the calculated estimation is slightly ahead of the developmental stage observed in other early *Homo* specimens (here by 6.5 months at most), yet this is may be accounted for by the variability which is not captured here, and by the uncertainties related to our measurements (e.g., the virtual section does not pass through the dentine horn tip).

### Comparison of GAR IVE with teeth from modern-day AI patients

**Figure 5:**
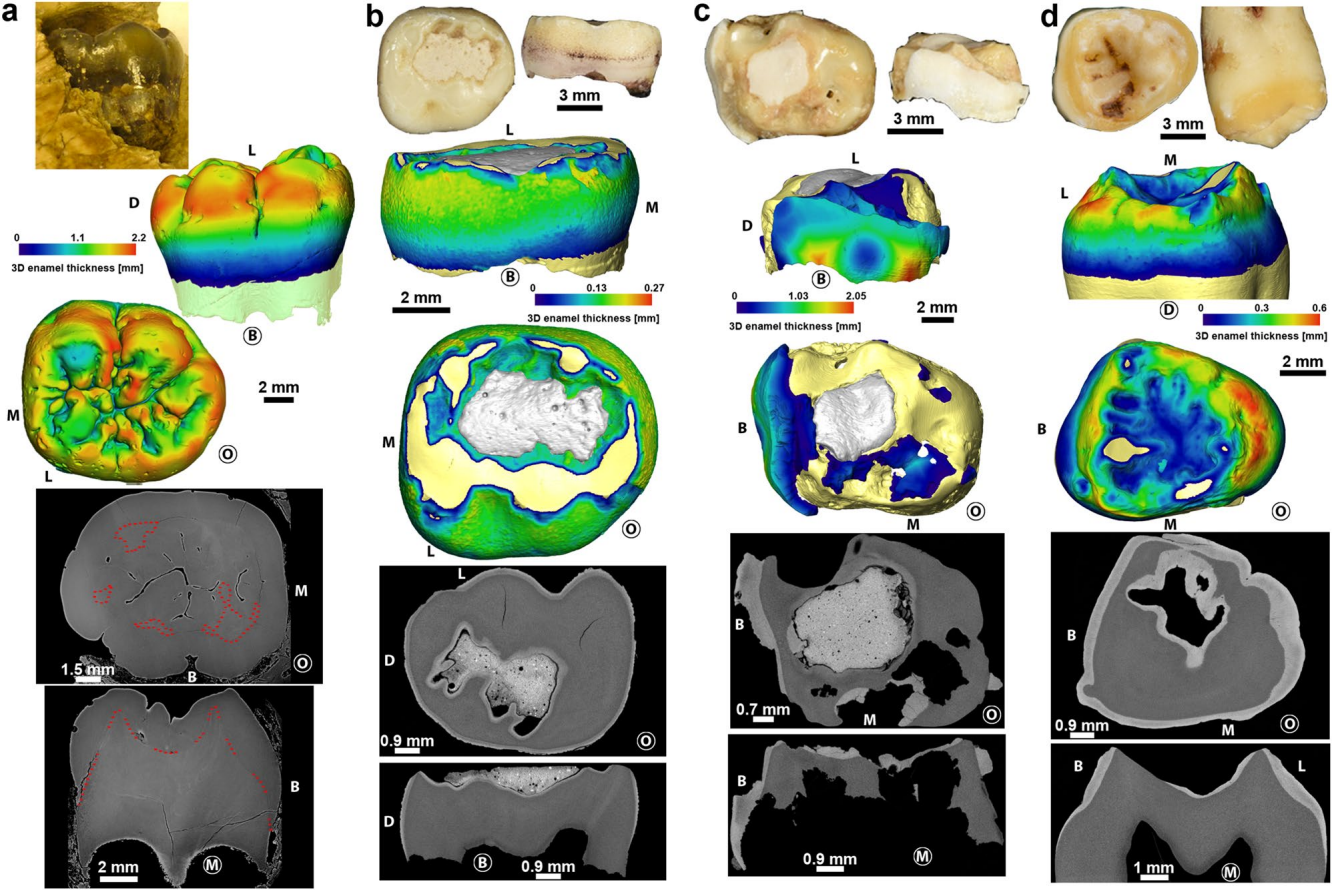
Comparison of the GAR IVE LRM1 (**a**) with three modern human teeth affected by different forms of amelogenesis imperfecta (**b**–**d**; see Supplementary Text S4for details). Photographs (top), 3D enamel thickness maps (middle) and 2D virtual sections (bottom) show that despite the loss of contrast, GAR IVE has a normal enamel thickness and morphology. Anatomical orientation is shows using ‘M’ for mesial, ‘D’ for distal, ‘B’ for buccal, ‘L’ for lingual and ‘O’ for occlusal. The side of the tooth in front view is circled.

Among the three clinical cases of molars affected by AI, two have maximal enamel thickness values considerably smaller (0.3 mm and 0.6 mm, respectively; Fig. 5b and d.) than that of GAR IVE (2.2 mm; Fig. 5a). The third comparative sample has retained negligible portions of thick enamel in the cervical region (2.05 mm; Fig. 5c). Beyond being remarkably thin, the enamel of this tooth (Fig. 5c) is also considerable brittle and has been fractured during its time in occlusion. The enamel surface of the deciduous molar in Fig. 5b shows extensive and shallow pitting. Both deciduous molars (Fig. 5b and c) have underwent some restoration treatment in their occlusal basin. Overall, this contrasts with the enamel of GAR IVE which is exempt of any fracture or chipping (Figs. 4, 5a and Supplementary Fig. S19).

### A taphonomic perspective to study a human remain

**Figure 6:**
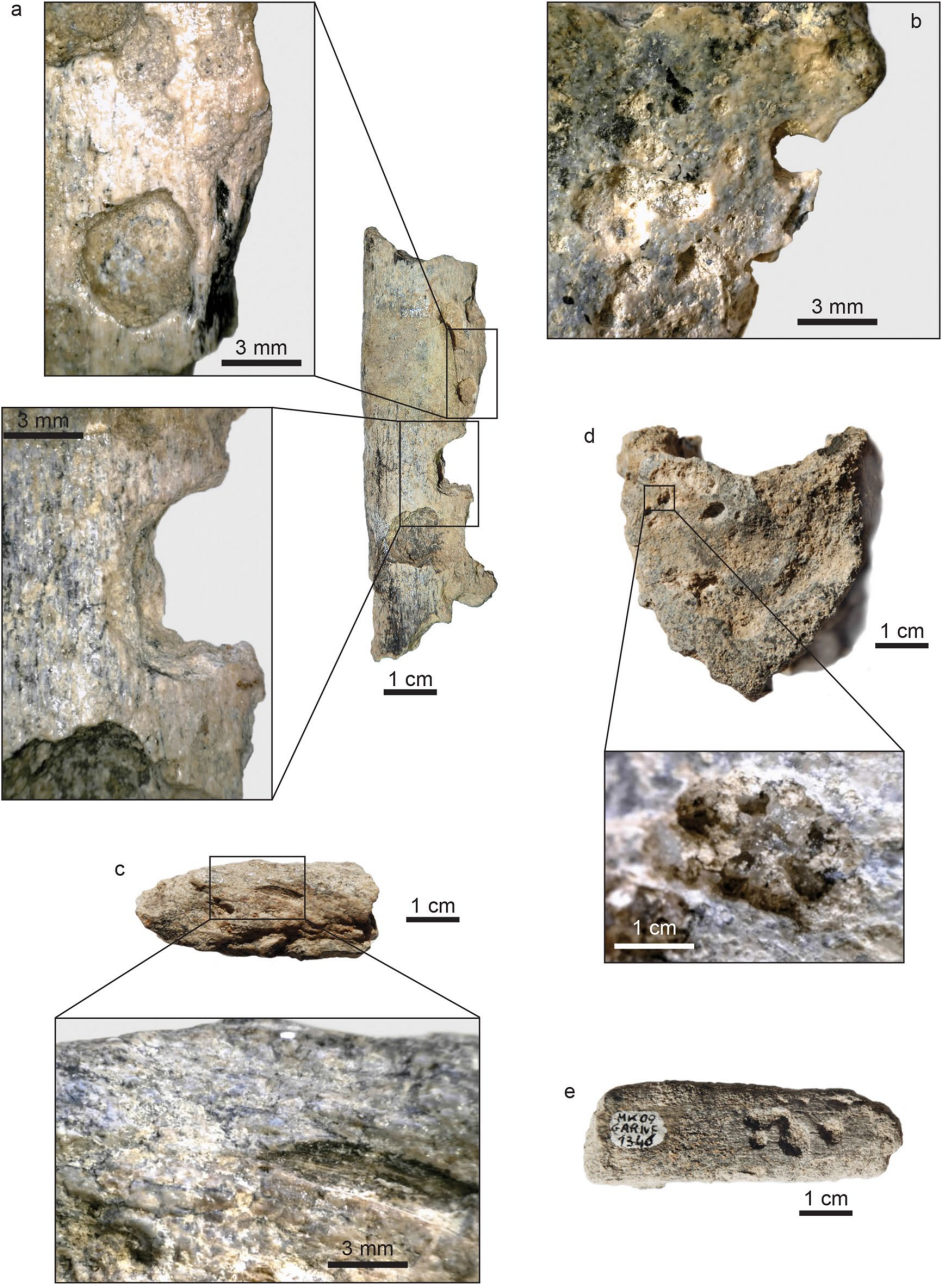
High quality photographs of faunal bone specimens from Garba IVE showing typical modifications left by dermestids. (A) Garba IVE 05-40, Garba IVE 05-57, Garba IVE 05-59, Garba IVE 05-32, Garba IVE 09-1346.

The largest bone loss on the buccal aspect of this right mandibular fragment is a hole which coincides with the anatomical position of the mental foramen (Figs. 2, 4). This cavity measures 5.5 mm in depth, 8.02 mm in outer width, from 3.4 to 4.1 mm in inner width and has a 7.51 mm-long tail on the outer bone surface. However, the fossil had been broken at the time of discovery, and curatorial activity led to some misalignment affecting the outline of this feature. Twenty-two faunal specimens of the Layer E also show evidence of perforations and characteristic damages (i.e., ~ 10% of the identified specimens) (Fig. 6, Supplementary Text S2; Supplementary Tables S10 and S11). Accordingly, the same agent was probably at work on fauna and hominin remains. We explored the impact of dermestid beetles (i.e., necrophagous insects), which were found to be active at African Plio-Pleistocene sites such as Laetoli and Makapansgat^46, 47^. These insects produce various traces, like surface tunnels, pits, and bore holes^48, 49^. According to Parkinson^49^ the presence of a minimum of three different traces in a fossil assemblage can be used to demonstrate the implication of dermestid beetles. In the faunal assemblage of Layer E, a bone fragment in particular (Fig. 6) shows holes very close in outline and size to the one observed on the GAR IVE juvenile mandible, and thus complies with dermestid beetles activity^50^. Accordingly, dermestid beetles likely fed and developed on the carcasses of animals in close proximity to the bony remains of GAR IVE, and possibly bore pupation chambers into the hominin bone (Supplementary Table S10 and Supplementary Fig. S17), and substantially destroyed the trabecular bone. Following this hypothesis, the insects may have taken advantage of the pre-existing mental foramen, and considerably enlarged it (Figs. 2, 4, Supplementary Text S2).

**Figure 7:**
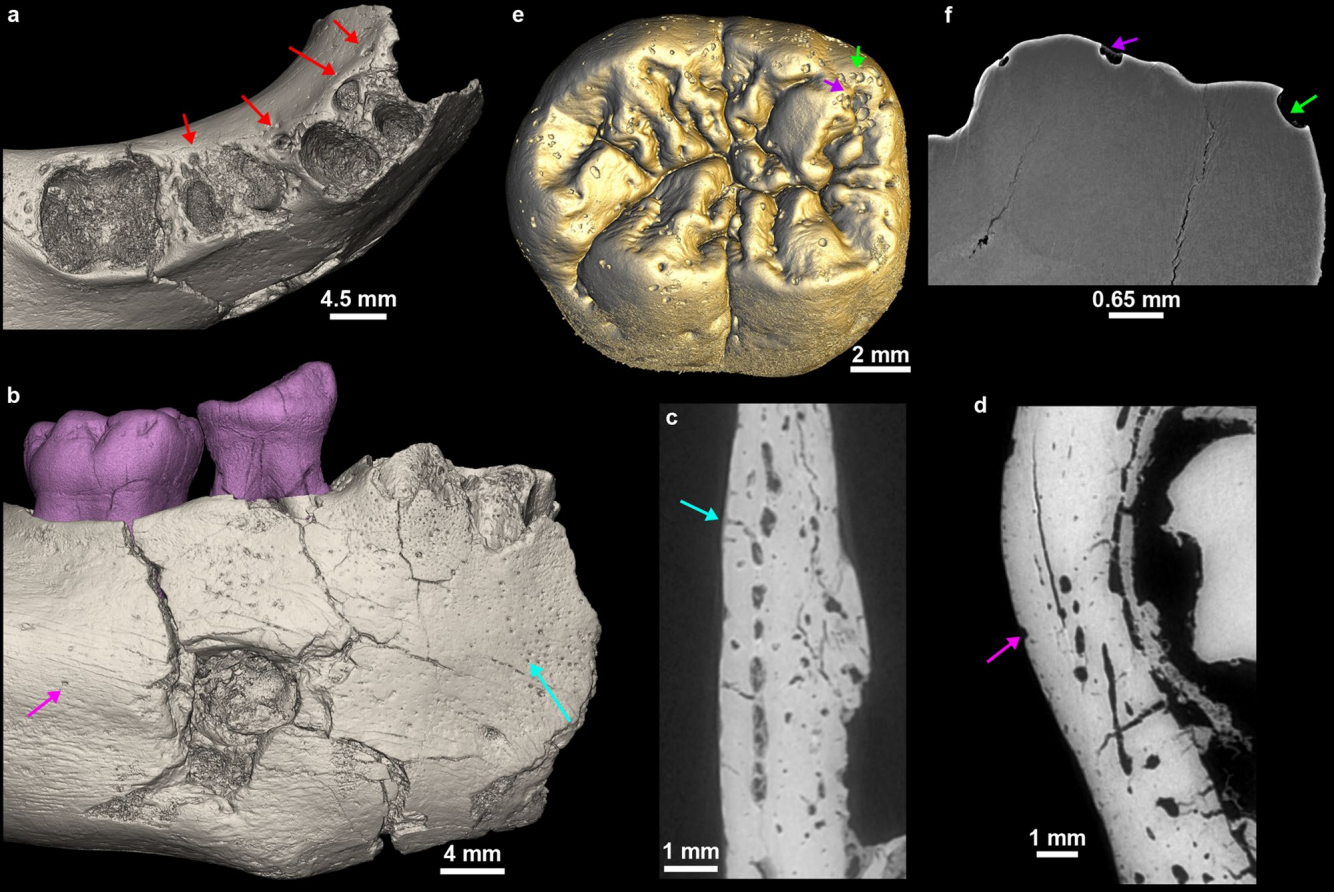
Variety of pits identified on GAR IVE related to the eruption of the permanent germs [gubernacular canals; red arrows on (**a**)], normal bone growth [Volkmann’s canals; blue arrow in (**b**) and (**c**)], and of taphonomic origin on the bone [pink arrow in (**b**) and (**d**)] and the teeth [arrows in (**e**) and (**f**)].

Relatively large cavities are restricted to the lingual aspect of the surface of the alveolar bone for each erupted deciduous teeth (Fig. 7). These holes are identified as the openings of the gubernacular canals which constitute a guide for the eruptive pathway of the developing permanent tooth germs^51–55^. These gubernacular foramina observed on GAR IVE have a circular diameter ranging from 0.75 mm to 2.46 mm, which is consistent with the standard values^56^. This normal and developmental structure exists in all vertebrates. Smaller pits (~ 0.15–0.20 mm in diameter) are distributed close to each other on the anterior portion of the mandibular corpus (Fig. 7). The holes are more densely distributed on the buccal aspect than on the lingual side of the bone, close to the anterior border. As seen in 2D virtual sections of the GAR IVE mandibular bone, these holes communicate with larger canals of the vascular system. These holes are foramina called Volkmann’s canals which are related to hypervascularisation occurring during normal bone growth and are regularly seen in juveniles^57–59^.

**Figure 8:**
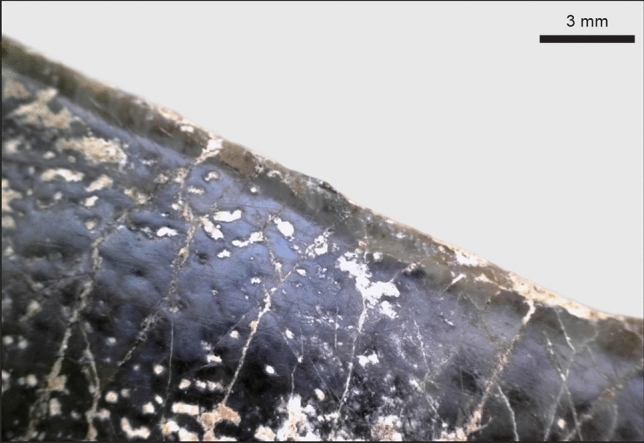
Close-up on the enamel surface of a *Hippopotamus* canine from Garba IVE, showing pits.

Small pits (~ 0.5 mm in diameter) can also be observed on the teeth and bone of GAR IVE (Fig. 7). They differ from both the previously described gubernacular canals’ openings and Volkmann’s canals on the bone. Pits can be observed mainly on the enamel surface of the GAR IVE LRM_1_. The deciduous molars are very slightly affected with only a couple of pits scattered on the outer enamel surface, and even on the exposed root dentine (Figs. 2, 7). These pits are strictly absent from the LRP_3_, LRP_4_ and LRC_1_ tooth germs, which are enclosed in the alveolar bone and therefore well protected from taphonomic processes. In sharp contrast, both the erupted deciduous teeth, the LRI_2_ and the LRM_1_, which were partially uncovered post-mortem as a result of a symphysis and ramus fracture, were exposed openly to taphonomic agents. The pits scattered on the bone surface have a more random distribution and are mostly restricted to the posterior half of the mandibular corpus. Interestingly, some of the animal bones and teeth of Layer E also display similar pits. The most striking example is a hippopotamus canine with noticeable pits all over its enamel surface (Fig. 8). Therefore, the same taphonomic agent may be held responsible in the case of both hominin and animal remains.

On the lingual side of the mandibular corpus of GAR IVE, four striae point to teeth sharpening by a small rodent (Supplementary Fig. S15), while the posterior portion of the buccal side shows traces of slight weathering (Supplementary Fig. S16). This is further evidence that the corpse of GAR IVE laid exposed and eventually decayed before being buried by alluvial deposits.

These additional surface features cannot be explained either by a pathological condition or by anatomical development. The characteristics of the general environment during fossilization, i.e., taphonomy, were thus considered as potential explanatory variables. They were clarified by the reconstruction of the sedimentary sequence of the Garba IV site where the fossil was unearthed (see Supplementary Text S3 for details). Garba IV is located in a volcanic area which was active during the Early and Middle Pleistocene. In this volcanic environment, chemical corrosion due to water acidity is better accounted for as a general cause of dental tissue alteration (see Supplementary Text S3 for details). In virtual 2D sections, the edges of the pits observed on the GAR IVE teeth appear sharp and acute (Fig. 7f), reminiscent of, and likely to be, vacuoles of dissolution.

## Discussion

Reaching a palaeopathological diagnostic on fossil remains often reveals challenging, even more so as the latter ones are usually fragmentary and damaged. Yet, this has always been attempted, ever since the nineteenth century, with mixed results, e.g., the Neanderthal specimen from Feldhofer Grotte^60^. Trinkaus^40^ has recently reviewed published palaeopathological cases, and mentioned GAR IVE and its putative AI as a likely cause for early death. The diagnosis of AI in GAR IVE was based on general enamel dysplasia, the abnormally severe attrition of the deciduous first molar, wrinkling and additional cuspules on the occlusal surfaces of the molars, enamel defects (i.e., shallow pits), as well as reduced enamel radio-opacity^19^. In agreement with Zilberman et al.^19^, Trinkaus^40^ further concluded that GAR IVE had suffered from restricted functional masticatory capacity resulting from AI, which induced malnutrition around weaning age.

Following Zilberman et al.^19^ ‘s diagnostic, GAR IVE would most likely have been affected by the AI variant called “hypoplastic, pitted autosomal dominant” Type IA as defined by Witkop Jr.^33^. In this variant, enamel may be totally absent or often fragile and significantly thinner to the point of breaking and eventually being totally lost^32, 35, 61^. The enamel surface is often rough, smooth or pitted. This is not the condition observed in GAR IVE: neither preserved deciduous nor permanent teeth are brittle or crumble, partly due to their normal enamel thickness (Fig. 5a, Supplementary Figs. S1 and S19). The inspection of the synchrotron µCT data confirms that the crowns are notably exempt of internal cracks (Figs. 2, 4, Supplementary Fig. S19). This seems to rule out the accumulation of fractures during life, and therefore contributes to the excellent fossilization of the enamel. Only a few cracks could be seen in the roots transversally and may likely have occurred after death. If the teeth had been made fragile during the infant’s life by an alteration of the enamel thickness and/or its microstructure, the tooth crowns would have very likely broken off as seen in modern clinical cases^35, 36, 62, 63^ (See Fig. 5). By definition, AI should affect enamel formation in all the teeth of both generations^32, 36, 38, 64^, which is clearly not the case in GAR IVE. Overall, some kinds of enamel defects may resemble these encountered in AI, although other causes may be identified: either pathological by a differential diagnostic^32^ or taphonomic^65^ as shown in GAR IVE. Taurodontism is often associated with the hypomaturation form of AI^31, 66^. The deciduous molars’ roots of GAR IVE are clearly divergent (Fig. 2), with a normally-sized pulp cavity (the chamber represents one third of the total pulp cavity in height while the root canals extend over the two thirds in height; Supplementary Fig. S1) and thus show no evidence of taurodontism. Accumulation of dental calculus has sometimes been reported in severe forms of AI^66^, which is not the case in GAR IVE, although it could have been lost post-mortem.

Severe attrition does not belong to the suite of manifestations defining the hypoplastic forms of AI, but rather in the hypocalcified and hypomaturation AIs. In the latter forms, enamel may prematurely chip away even before tooth emergence^35^, which is not the case in GAR IVE.

The extensive wrinkling of the occlusal surface of the GAR IVE LRM_1_ (Fig. 3) looks similar to that reported in recent modern humans, in which it is more common on the outer enamel surface than at the enamel-dentine junction, and also in the M_1_ than in the dm_2_^67, 68^. This morphology has also been observed in several fossil hominin taxa, among which East African robust australopithecines^69^. The occlusal wrinkling of the Omo 427 LRM_2_ (Fig. 3) resembles that of GAR IVE, while it is even more pronounced in the Omo 136–1 LLM_3_. The LRM_2_ of *Australopithecus anamensis* KNM-ER 34725 T is also very similar to GAR IVE (See Fig. 2 in^70^). This supports the hypothesis that this trait is not pathological and rather results from the retention of a primitive morphology in healthy modern humans, and in GAR IVE.

Concerning the pits mainly observed on the enamel surface of the GAR IVE LRM_1_, no comparable dental defects could be found in the medical literature. Towle and Irish^71^ describe that *Paranthropus robustus* molars show the most frequent occurrence of pitting enamel hypoplasia (PEH) than any other extant Primates studied. They further notice that the pits are more frequent on deciduous than on permanent teeth, the anterior teeth not being affected. Towle and Irish^71^ make a correlation between this PEH and the pits observed in modern humans affected by AI, and thus propose that these pits have a genetic origin. If the defects observed on GAR IVE had a genetic and developmental origin, their edges—which were shown to be sharp (Fig. 7)—would have been eroded during the life of the infant due to contact with the saliva and the food items processed in the mouth. Therefore, these enamel alterations may have most likely occurred after death. Additionally, several kinds of pits were also observed on the bone surface, and were interpreted in terms of bone growth (Volkmann canals), tooth development (openings of the gubernacular canals) and taphonomy (See Results).

Some of the clinical criteria commonly used to diagnose AI cannot be reliably used on fossil dental remains, such as the radio-opacity and the coloration of enamel, due to taphonomic processes. The most obvious of them concern changes in enamel colour, which is often used for a differential diagnosis with fluorosis together with enamel flecking and hypoplasias^32^. In fossils, a discolouration of enamel may more likely be related to taphonomic processes^65^, as is the case for the very dark enamel of GAR IVE. The argument for the reduced radio-opacity^19^ is tricky to consider in fossil teeth as diagenesis may well have altered the original composition of the tooth microstructure. In hypoplastic forms of AI, the contrast between enamel and dentine appears normal radiographically^72^. In GAR IVE, taphonomic processes inducing a remineralization may have altered the coefficient of attenuation of the dental hard tissues to X-rays, thus obscuring its fine enamel microstructure (Figs. 5a, 7f, Supplementary Figs. S1, S19). Only a couple of accentuated lines could be observed in the cuspal enamel of the LRP_4_ (See Supplementary Text S1).

Other surface features attest that several taphonomic agents were at play, such as on the posterior portion of the mandibular corpus, where weathering affected the buccal side and rodents sharpened their teeth on the lingual side. The buccal aspect of the mandibular corpus shows a substantial bone loss at the location of the mental foramen. Zanolli et al.^21^ interpreted this cavity as a carnivore tooth mark. The breakage pattern and fissures observed on GAR IVE do not match that resulting from a carnivore bite. To note, that the lingual side of the right mandibular corpus lacks the mark of the counterpart tooth, which may not have systematically imprinted the bone anyways. The size of the hole considerably exceeds that of puncture marks produced by for instance hyenas (Supplementary Fig. S18). Importantly, the high resolution synchrotron scans reveal a virtual 3D negative imprint of the hole showing a convoluted shape, which is little compatible with the shape of a carnivore tooth (Fig. 4). Noteworthy, there are overall very few carnivores in the faunal record of Melka Kunture^24^, except the large civet *Pseudocivetta ingens* in Layer E of Garba IV^73^ (Supplementary Table S11).

Whether the ontogenetic stage “childhood” has emerged from the *Homo* lineage at ~ 1.8 Ma is still highly debated. There are currently too few juvenile early *Homo* fossils recovered with well-preserved dental and skeletal remains to assess and discuss the pattern and timing of their dental development and skeletal maturation^5^. However several lines of evidence based on dental development studies point to a lack of childhood in early *Homo*. Indeed dental examination of the Nariokotome boy (KNM-WT 15,000, 1.5 Ma) showed that this juvenile, deceased at 8–9 years of age, presented an advanced skeletal and dental maturation suggesting that growth was rapid, with the lack of a slowdown growth characteristic of childhood^11^. KNM-ER 1507 and KNM-ER 820 (See Supplementary Figs. 9 and 10) represent two young *H. erectus* s.l. with an estimated age at death of 5 years^74, 75^. Both specimens are more advanced than *P. boisei* specimens in terms of calcification stages^74^. This observation is interesting in light of our comparison of the GAR IVE tooth calcification stages with other fossil hominins, and of the rough estimation of its age at death in comparison with DNH 35 (see Results). The comparison of GAR IVE with modern human equivalents as described by AlQahtani and colleagues^42^ show a tremendous variation in mosaic among tooth type (See Supplementary Text S1 and Table S1), likely reflecting a variability in the time of initiation of the germs, and of their rate of calcification. The scoring of each of the GAR IVE teeth after AlQahtani and colleagues^42^ provides a range of 3 to 8 years. This is consistent with the results of the Bayesian statistics performed in Zanolli et al.^21^, where the calcification sequence of GAR IVE was found in four modern human children aged 2.67, 3.92, 4.54, and 6.42 years. KNM-ER 820 (mandible) has the permanent first molar in occlusion and already worn^74^ confirming a rapid growth in this species. Early *Homo* at around 1.5–1.8 Ma would not have experienced any significant period of slow somatic growth between the end of infancy and the juvenile stage^1, 11, 74–76^. Thompson and Nelson posit that childhood bears a cost to the group of hominins^5^. Although having a stage of childhood decreases child mortality and increase the fertility rate of the mothers, the pressure increases on the whole group to care for the offspring in terms of provisioning and protection during their least productive years of life^5^. Since taphonomy has obscured most the dental microstructure in the GAR IVE teeth, a fine quantitative documentation of the pattern and timing of its dental development remains yet inaccessible. The accentuated lines occurring in some of its tooth germs could have been imprinted by strong stress events occurring before death. It remains however totally speculative to propose that these repeated stress events may have had an impact on the chances of survival of the child, or its health and growth. The circumstances of the death of GAR IVE at a very early stage of its life – presumably before 3 years of age – remains unelucidated. However, our findings proposing that GAR IVE has not suffered from AI restore the status of this early *Homo* infant as not being affected by a pathology which could have been lethal. The tooth morphology of GAR IVE can thus be reliably included in future comparative studies, such as for refining its taxonomic status.

## Conclusion

High-resolution synchrotron imaging allowed reassessing the diagnosis of AI in GAR IVE. Among the arguments raised by Zilberman and colleagues^19, 30^, several cannot be grounded in GAR IVE. We demonstrated that some features interpreted as symptoms of AI are normal anatomical features related to bone growth (i.e., Volkmann’s canals) and tooth eruption (i.e., gubernacular canals), while others are resulting from post-mortem process. In general, there is no evidence of a pathological enamel condition in GAR IVE, and all these observations invalidate the diagnosis of AI in this early *Homo* juvenile. GAR IVE can thus be reliably used in future comparative studies as one of the rare early *Homo* infant specimens preserving a partial mixed dentition.

Overall, our results suggest caution when diagnosing a rare genetic disease on a fossil remain, and considering its implications on our understanding of hominin life history and consequently, on human evolution. Fossil human remains gain to be investigated using an integrative approach, combining different fields of expertise, such as taphonomy, environmental reconstruction, archaeological and geological context, high-resolution imaging techniques and/or a clinical approach, whenever needed.

## Methods

The GAR IVE mandible is kept in the National Museum of Addis Ababa (Ethiopia) together with the archaeological and palaeontological collections from Garba IV and all other sites of Melka Kunture. ARCCH (Authority for Research and Conservation of the Cultural Heritage) provided the permit for the temporary export of the fossil to be scanned in Grenoble (France) at the European Synchrotron ESRF.

Since the variously-sized and -shaped surface morphology on the GAR IVE teeth had been interpreted as abnormal and lead to reach the general diagnosis of AI, we explored the tooth surface features. Concerning the developmental alterations that AI produces, the analysis of the enamel microstructure is especially valuable. Indeed, dental hard tissues record their own growth and maturation as well as stressful events experienced by the organism^11^. In the enamel, stress events may manifest as accentuated lines which remain non-specific^12–14^. To note that birth is recorded by the neonatal line often identifiable in the enamel and dentine of in deciduous teeth and the mesial cusps of the permanent first molar which start mineralizing *in utero*^15, 16^.

To access the tooth and bone microstructure of GAR IVE, we scanned the mandible and its dentition at high-resolution using synchrotron propagation phase contrast micro-computed tomography (PPC-SR-µCT). The scans were acquired by Dr. Paul Tafforeau at the ESRF (Grenoble, France) on the ID 19 beamline. The technical parameters used for the data acquisition are listed in detail in Supplementary Text S4. Two- and three-dimensional techniques of virtual histology were used in VG Studio MAX 3.2 (Volume Graphics, Heidelberg, Germany) to explore the preservation of bone and dental tissues^77^.

To reassess the diagnosis of AI in GAR IVE, and further study the 3D enamel thickness distribution in its LRM1, we aimed to access clinical extractions of teeth from patients diagnosed with AI. Despite a wide query in clinical institutions in several countries, we could eventually only access three teeth. The first tooth (“AI_1”, (d) in Fig. 5), an ULM3, was collected after extraction indicated by an orthodontic treatment, at the “Centre de référence des maladies rares du métabolisme du phosphore et du calcium” Service d’Odontologie, Hôpital Bretonneau, AP-HP, Paris, France. All teeth were collected with informed and written consent from the patients according to ethical guidelines set by the French law (Loi Bioéthique n°2004–800) and with special ethical authorization for brittle teeth (from the “Comité d’Evaluation de l’Ethique des projets de Recherche Biomédicale (CEERB) Paris Nord” Institutional Review Board -IRB 00,006,477- of GH Nord Université de Paris, AP-HP) and tissue and cell banking agreements (n°DC-2009–927). This female patient was genotyped and was shown to be affected by a mutation on AMELX, which rather argues in favour of an hypoplastic form of AI. The two other teeth are from an historical sample curated in the “Anatomical Collection” of the Department of Oral and Maxillofacial Sciences at the University of La Sapienza, Rome, Italy. Besides the record of that this is a case of AI, there is no further information regarding the deciduous molar “AI_2” ((b) in Fig. 5). According to the historical records of the anatomical collection, the third tooth (“AI_3”, (c) in Fig. 5) is an ULdm2 extracted from an 11 year-old girl, who was clinically diagnosed AI. Although there is no more specific information, and due to its brown enamel, it is most probably the hypocalcified form of AI, and potentially the hypocalcified 3A variant after Witkop Jr. (1988). To note that the patients were diagnosed with different forms of AI, with various degrees of certainty and amount of information. We decided to report on the few teeth we had access to, to show a variety of manifestation of putative forms of AI in modern humans. The teeth were scanned on a custom made Diondo D3 µCT-scanner in the Department of Human Evolution, at the MPI-EVA (Leipzig, Germany), and the reconstructed µCT data were analysed in Avizo 7.0 (FEI) (Supplementary Text S4 for further details).

Faunal remains were examined to determine what taphonomic process have been a play at the site, and whether they could have produced the features observed on GAR IVE (e.g., pits on the outer enamel surface) and interpreted as being related to the AI pathology. Taphonomy encompasses the characteristics of the general environment during fossilization. Our approach involved the reconstruction of the palaeonvironmental context of Garba IVE, which is based on the detailed analysis of the stratigraphic sections carried out in 2013 and 2017. Sedimentary features at the meso- and micro-scale (grain size, structure, texture, unconformities) were studied for the reconstruction of the depositional processes, volcanic facies and for correlation with the stratigraphic sections along the Garba and Gombore gully. The results were eventually correlated with the dataset of Raynal and Kieffer^78^. The taphonomic observations conducted on GAR IVE and the associated faunal remains were performed using 10 ×-20 × hand lenses and a Dino-Lite Edge 5MP, at the National Museum, in Addis Ababa, Ethiopia.

## Supplementary Information

Supplementary Information.
