# Supplementary material for: Insights into the palaeobiology of an early Homo infant: multidisciplinary investigation of the GAR IVE hemi-mandible, Melka Kunture, Ethiopia
Source: Sci Rep. 2021 Nov 29;11:23087. doi: 10.1038/s41598-021-02462-1 (PMC8630034; doi:10.1038/s41598-021-02462-1)
Supplement: Supplementary file 1 — Supplementary Information. [file 41598_2021_2462_MOESM1_ESM.pdf]

SUPPLEMENTARY INFORMATION

Insights into the palaeobiology of an early *Homo* infant: multidisciplinary investigation of the GAR IVE hemi-mandible, Melka Kunture, Ethiopia.

A. Le Cabec, T. Colard, D. Charabidze, C. Chaussain, G. Di Carlo, S. Gaudzinski-Windheuser, J.-J. Hublin, R. T. Melis, L. Pioli, F. Ramirez-Rozzi, M. Mussi.

Contents

1. *Estimating the age of the stress events in LRP4 enamel (Suppl. Text S1; Suppl. Figs. S1-14; Suppl. Tables S1-9).* .... *p. 1*
2. *Taphonomic investigation of the early* Homo *GAR IVE mandible and associated faunal remains (Suppl. Text S2, Suppl. Figs S15-19, Suppl. Tables S10-S12)* .... *p. 14*
3. *Reconstruction of the palaeoenvironment (Suppl. Text S3).* .... *p. 21*
4. *Technical parameters for the µCT data acquisitions (Suppl. Text S4; Suppl. Tables S13-15)* .... *p. 22*
5. *Cited references* .... *p. 25*

1. Estimating the age of the stress events in LRP4 enamel. (Suppl. Text S1)

1.1. Estimating GAR IVE's age at death using modern standards

Virtual developmental sections were recorded in each tooth to assess its stage of formation (e.g., [1]). Since the inner structure of the teeth was obscured by taphonomic alterations, the EDJ was drawn in colour after close inspection of the scans to facilitate the assessment and the scoring of the developmental stages (Suppl. Fig. S1).

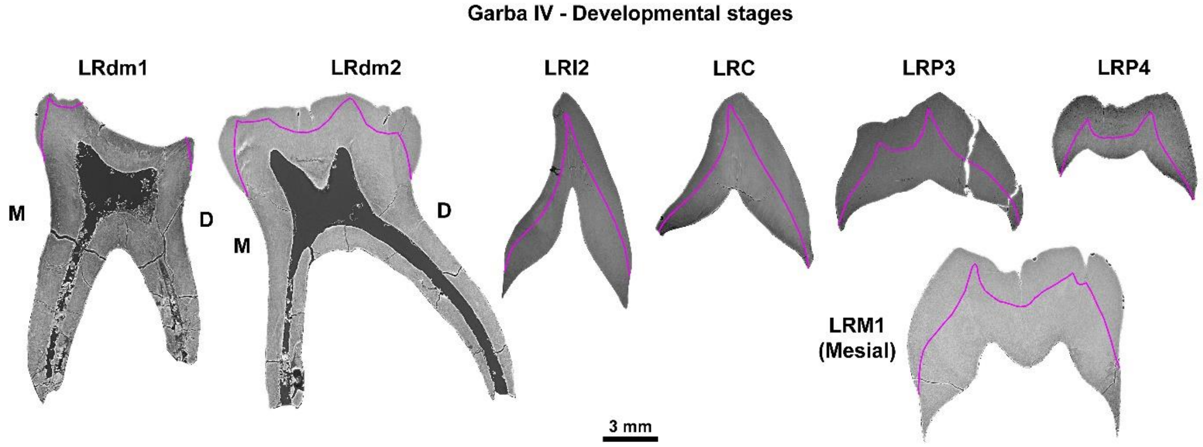

Supplementary Figure S1 – Virtual developmental 2D sections through the GAR IVE deciduous and permanent teeth. Due to the lack of contrast, the EDJ was delineated in pink to facilitate the visualisation of the enamel and dentine thickness.

To note that the I_2 has developed 1.5 mm of root on the lingual aspect, and ~2 mm of root on the buccal aspect, the P_3 has formed 0.6 mm on both sides, and the M1 has

formed 2.6 mm of root on its lingual aspect, for 3.8 mm on the lingual side of its mesial section.

1.1.1. Recent modern humans

Based on radiographs, Condemi[2] had estimated GAR IVE's age to fall between 3 and 4 years. Zanolli et al.[3] refined this estimation at 2.5-3.5 years.

After inspection of the PPC-SR µCT data (Suppl. Fig. S1), the calcification stages were scored and corresponding median ages were listed per tooth type after the modern human atlas built by AlQahtani et al.[4] (Suppl. Table S1).

Mandibular tooth type	dm1	dm2	I2	C	P3	P4	M1
Score for tooth formation stage (after Fig. 1 in AlQahtani et al.[4])	Ac to Res1/4	At least Rc	R1/4	~CrC	Ri	Cr1/2	R1/4
Median corresponding age (years)	8‡	3*	5.5†	5.5†	7.5‡	5†	5.5†

*: from Table 4 ; †: from Table 5; ‡: from Table 6 in AlQahtani et al.[4].

Supplementary Table S1 – Scoring of the calcification stages of GAR IVE, and estimated corresponding age in modern human standards after AlQahtani et al. (2010)[4].

The overall pattern of GAR IVE would be equivalent to **4.5 years** in modern human equivalent after Fig. 6 in AlQahtani et al. (2010)[4] (Suppl. Fig. S2)

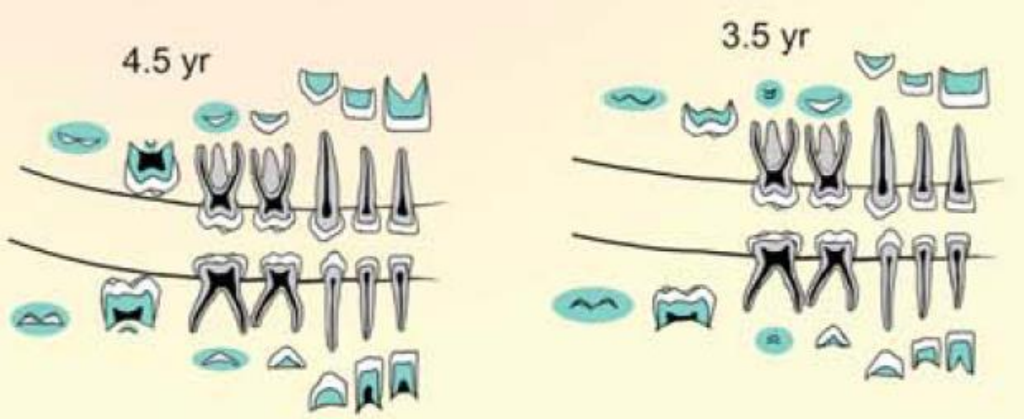

Supplementary Figure S2 – Modern human developmental stages closest to GAR IVE, with a higher match with the 4.5 years schematic (modified after Figure 6 in AlQahtani et al., 2010 [4]).

1.1.2. Chimpanzees

Kuykendall[5] designed a scoring system based on Demirjian and colleagues[6] for exploring chimpanzee dental development. From the collected data on these known age specimens, he built a regression model to calculate a developmental age. Kuykendall [5]'s equation is as follows:

$$Age = 0.078Dm + 0.002\ Dm^2 - 0.073 \pm 0.99$$

Where “Dm” is the sum of the individual scores for a quadrant of the mandibular dentition (in this equation, omitting the M3).

This scoring system is often used to estimate a chimpanzee-equivalent age for fossil hominins (e.g., [7,8]).

In GAR IVE, the LRI1 is not preserved but is assumed to be at least at a stage equivalent to that of the LRI2. The LRM2 is also missing, but since its initiation is likely very close in time to that of the LRP4, their developmental stage is assumed to be the same.

Tooth type (mandibular right quadrant)	I1	I2	C	P3	P4	M1	M2	Dm
Kuykendall (1996) score	5	5	4	4	3	5	3	29

Supplementary Table S2 – Scoring of the calcification stages of GAR IVE after Kuykendall (1996)[5].

The sum of these scores is Dm = 29 (Suppl. Table S2). This yields a “chimpanzee-equivalent” age of 3.87 years with a range of 2.88 – 4.86 years.

Yet, since Kuykendall[5]'s scoring was designed on radiographs and that we score on µCT data, the regression underestimates the stage of development of the germs. We apply the conversion system designed in Gunz et al. [8] on the above radiographic stages to take into account the µCT data:

	I1	I2	C	P3	P4	M1	M2	Dm
Scores on µCT	4.5	4.5	3.5	4.5	3.5	4.5	3.5	
Converted Kuykendall scores	4	4	3	4	3	4	3	25

Supplementary Table S3 – Scoring of the calcification stages of GAR IVE after converting the Kuykendall (1996)[5] scores from the use of µCT data after Gunz et al. (2020)[8].

The new sum of these converted scores is Dm = 25 (Suppl. Table S3), leading to a new age and range estimate : ***3.13 [2.14 – 4.12] years***.

1.2. Early hominins of similar dental developmental stage

Several early hominins show a stage of dental development similar to that of GAR IVE, some of which have a published histological age at death[7,9]. The stage of development of the M1 roots and of the P4 are of special interest.

-*P. boisei* KNM-ER 1477: 2.5-3.0 years [9].

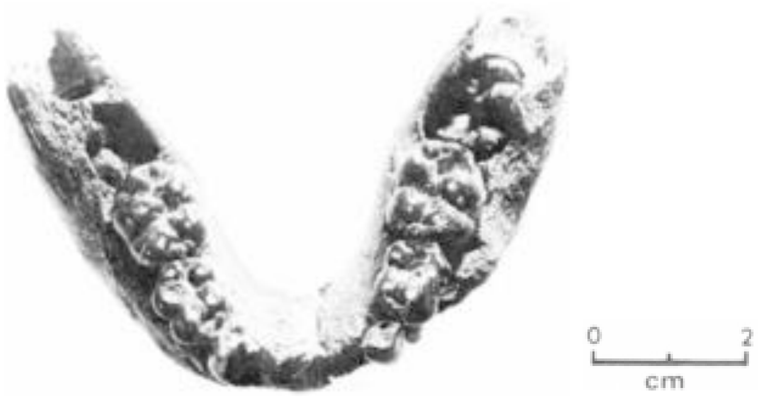

Fig. 1 Occlusal view of juvenile mandible KNM-ER 1477.

Supplementary Figure S3 – Photograph of the occlusal aspect of KNM-ER 1477 (After Fig. 1 in Leakey, 1973[10]). See Fig. 1 in Dean, 1987[9] for periapical radiographs of the developing teeth of KNM ER 1477.

KNM-ER 1477 (Suppl. Fig. S3, and Fig. 1 in Dean, 1987[9]) is slightly less advanced than GAR IVE as its M1 has achieved crown formation but has yet not initiated root formation. The C, P3 and P4 are still developing their crown, with P3 being more advanced than P4.

-*P. boisei* KNM-ER 812: 2.5-3.0 years [9].

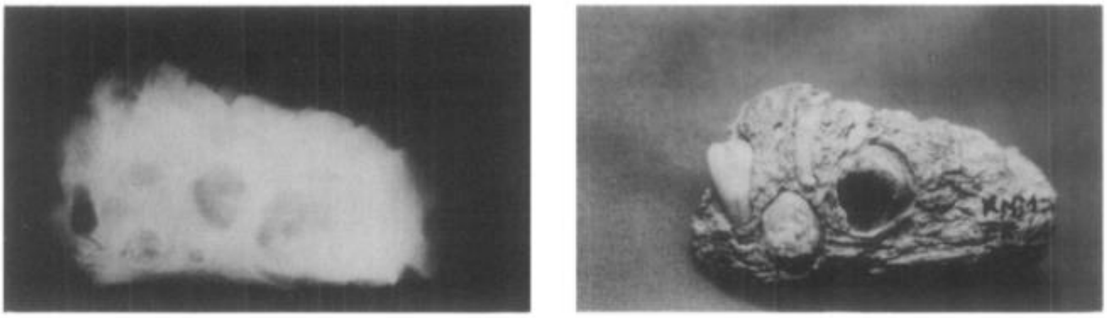

Figure 2. Radiograph of specimen KNM ER 812 together with a photograph of the specimen, (buccal aspect) that also demonstrates the exposed developing permanent teeth.

Supplementary Figure S4 – Radiograph and photograph of KNM-ER 812 (after Fig. 2 in Dean, 1987 [9]).

KNM-ER 812 (Suppl. Fig. S4) seems to overall follow the same pattern and timing of dental development. Besides the preserved roots of the deciduous canine and molars, I1 has completed its crown and has already formed ~2mm of root. The I2 is slightly less advanced. The canine crown is still developping. P3 and P4 are still forming their cuspal enamel. M1 has complete crown and started root formation.

-*P. boisei* KNM-ER 1820: 2.5-3.1 years [9].

In the left mandibular corpus of KM-ER 1820 (Suppl. Fig. S5), the I1 has formed >2mm of root, the canine and P4 crowns are developing, while the P3 has completed its cuspal enamel. The M1 has started forming its roots although the furcation level has not been attained yet. This makes it slightly more advanced than GAR IVE.

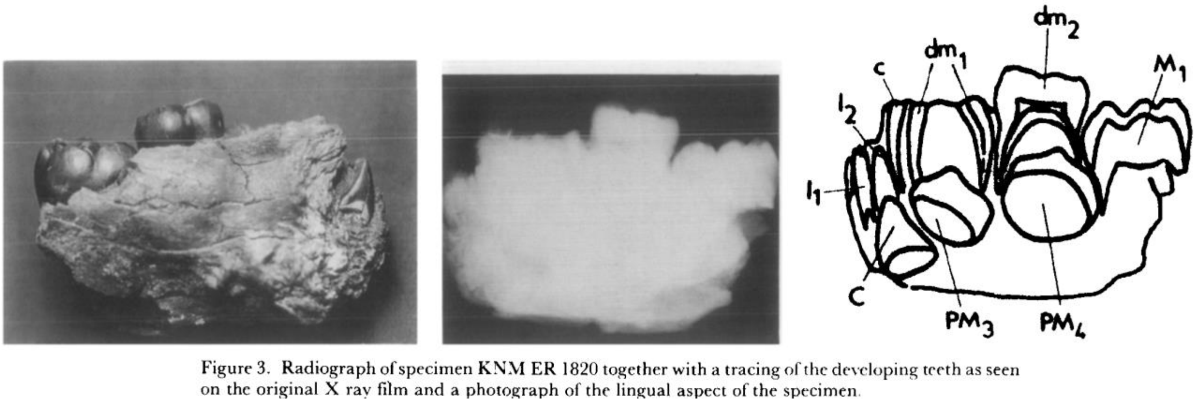

Supplementary Figure S5 – Photograph and radiograph of KNM-ER 1820 (after Fig. 3 in Dean, 1987 [9]).

- *A. anamensis* KNM-KP 31712: 2.8 years [7].

KNM-KP 31712 shows the same developmental stage as GAR IVE for both the permanent canine and M1 (Suppl. Fig. S6).

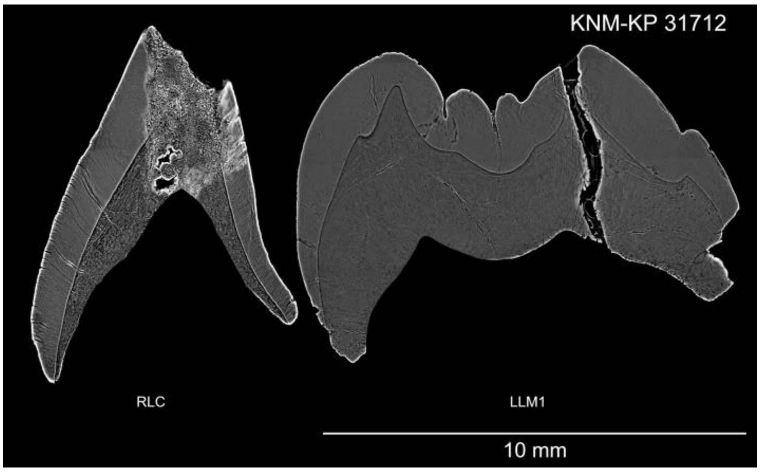

Supplementary Figure S6 –Virtual 2D section through the developmental plane the LRC and LLM1 of *A. anamensis* specimen KNM-ER 812 (after Fig. C in S1 file of Smith et al., 2015[7]).

- *P. robustus* SK 62: 3.12 years [7].

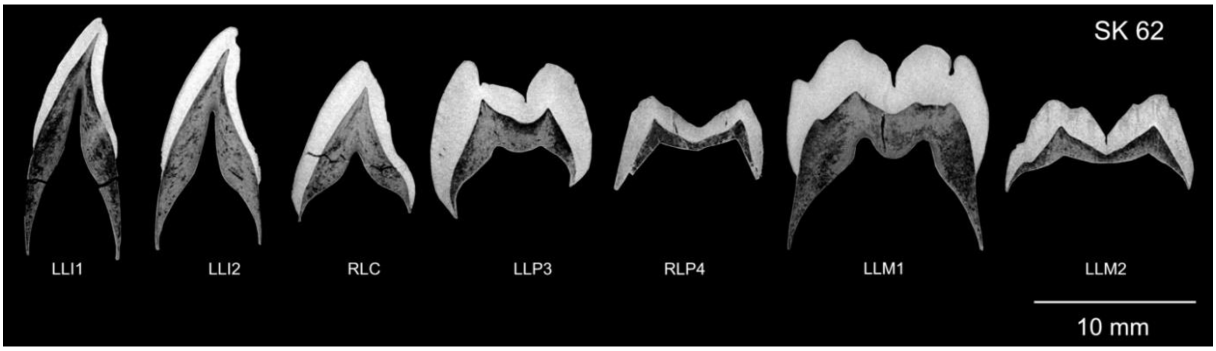

Supplementary Figure S7 –Virtual 2D section through the developmental plane the permanent tooth germs of *P. robustus* specimen SK 62 (after Fig. H in S1 file of Smith et al., 2015[7]).

Compared to SK62 (Suppl. Fig. S7), GAR IVE has a similar stage for its M1 and C. Yet, SK62's incisors are much more advanced (almost reaching half of the root formation). GAR IVE's P4 is more advanced.

-*P. boisei* OH 30: 2.7-3.2 years [9].

OH 30 consists in isolated teeth[9]: 3 deciduous teeth and 13 developing permanent teeth with virtually no root formed yet (no photo nor radiographs). The I1 is just complete while the I2 and C crowns are still forming. The M1s have completed crown formation and, in some places, 1 mm of root can be observed. The P3 and P4 have finished forming their cuspal enamel.

- *A. africanus* Sts 24: 4.35 years [7].

GAR IVE is developmentally less advanced than Sts 24 (Suppl. Fig. S8), especially regarding its M1 root which has not formed until the furcation level. In Sts 24, the C, P3 and P4 are at similar stages than in GAR IVE. To note that GAR IVE's I2 is more advanced in having initiated its root formation.

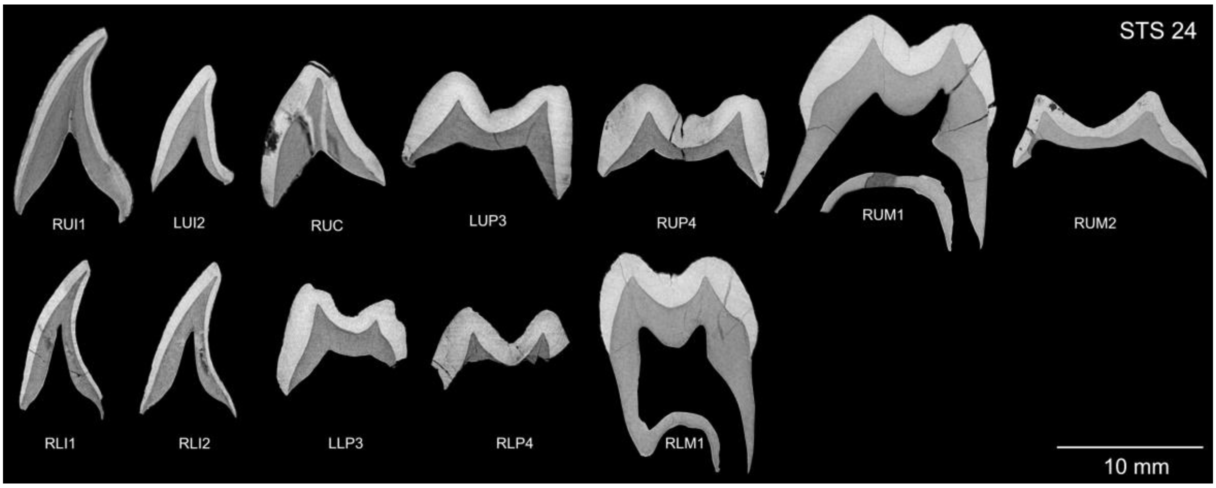

Supplementary Figure S8 –Virtual 2D section through the developmental plane the permanent tooth germs of *A. africanus* specimen Sts 24 (after Fig. F in S1 file of Smith et al., 2015[7]).

- Early *Homo* KNM-ER 820 : 5.3 years[11], and KNM-ER 1507[9].

For both early *Homo* specimens, no histological age at death was estimated, yet, base don perikymata counts on an I_2 and an estimated age of initiation, KNM-ER 820 was estimated to be 5.3 years[11]. The following descriptions follow the data provided by Dean (1987)[9].

KNM-ER 820 (Suppl. Fig. S9) is a fairly complete mandible with a mixed dentition. The four deciduous molars are preserved although worn because in functional occlusion. The deciduous canines are slightly less well preserved. Among permanent teeth, 6 are erupted, 8 are still in their crypt. The central incisors are worn to the dentine with root apices still open. Both I_2 show minimal wear and also have their root apex open. The canines and the premolars are at a similar calcification stage, with 3.6 mm and 4-5 mm of root developped, respectively. The M_1s are in occlusion, with the dnetine horns of the mesio-buccal cusp exposed, and the root apices open. The M_2 are not emerged although they have completed their crown and started root formation. The M3 are not formed yet, i.e., the crypt is visible but no cusp could be identified on the radiographs.

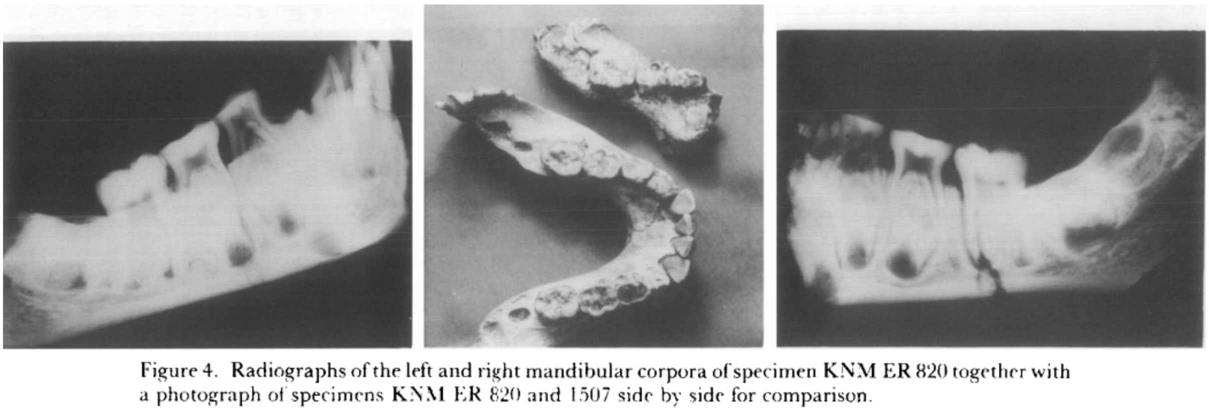

Supplementary Figure S9 – Radiograph of the right (left) and right (left) side of the mandible of early *Homo* KNM-ER 820. Photograph (middle) of this specimen besides the early *Homo* fragmentary mandible KNM-ER 1507 (after Fig. 4 in Dean, 1987[9]).

KNM-ER 1507 (Suppl. Fig. S10) is a fragment of left mandible preserving 3 erupted teeth (dm_1, dm_2, and M_1) and 4 developping teeth in their crypt (C, P_3, P_4, M_2). The canine has 2.5 mm of root formed, the M_1 is in occlusion, already slightly worn and its root apices are still open. The P_3 has completed crown formation and has developped 2 mm of root. The P_4 is at a slightly less advanced stage of development. The M_2 is at a stage of calcification similar to the P_3, with 1.5-2 mm of root formed.

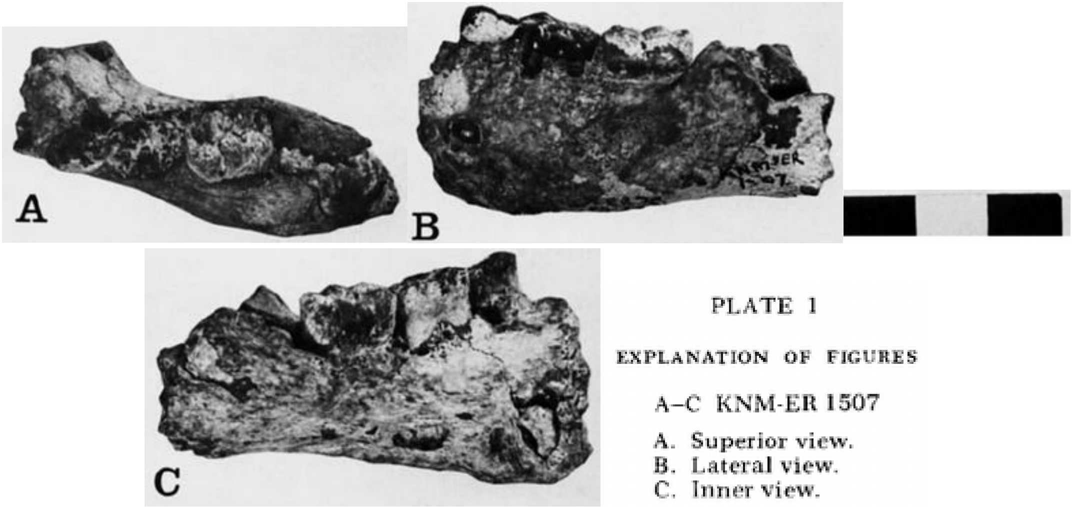

Supplementary Figure S10 – Photograph of the occlusal (A), buccal (B), and lingual (C) aspects of early *Homo* KNM-ER 1507 (After Plate 1 in Leakey and Wood, 1974[12]). See Fig. 5 in Dean, 1987[9] for a radiograph of the developing teeth of the specimen.

KNM-ER 1507 and 820 have their M_2 and premolars developping at the same pace, although KNM-ER 820 has a slightly more advanced M_2. Dean (1987)[9] also highlight that both early *Homo* are developmentally similar to four *Paranthropus* specimens which are OH30, KNM-ER 1477 (Suppl. Fig. S3), KNM-ER 812 (Suppl. Fig. S4), and KNM-ER 1820 (Suppl. Fig. S5). Yet, Dean (1987) noticed that the early *Homo* infants are both 'dentally more mature than [these] specimens of *P. boisei* described [as] being closer to 5 years of age'[9]. (Words in square brackets are our modifications).

Both early *Homo* specimens are developpementally older than GAR IVE (Suppl. Figs S9 and S10).

1.3. Estimating the age of stress events in the enamel of the LRP4

1.3.1. Time of P4 initiation in early *Homo*

In hominids, both M2 and P4 initiate together ([4,5,7]; see Sts 24 in Suppl. Fig. S8).

Dean and Smith[13] show that the Sangiran S7-37 P^4 and the KNM-WT 15000 M^2 initiate at about the same age, shortly after 2 years. Dean[9] described the stage of development of postcanine teeth in juvenile early *Homo* KNM-ER 820 and KNM-ER 1507 (see Suppl. Figs S9 and S10), and observed that, in each specimen, both premolars seem to parallel their M_2 in terms of developmental stage. Dean [14] further notice that M1 and I1 have coinciding calcification stages in *Paranthropus* and early *Homo* (See Fig. 2.5 in [14]). Interestingly in *Paranthropus robustus*, M2 also initiates shortly after 2 years of age (735 days for ML cusp of SK 62, and 823-888 days for the ML cusp of DNH 108; [7]). To note that *Australopithecus africanus* STS 2 has just fused both cusps in its ULP4 at the time of death at 2.52 years [7]. DNH 35, a South African early *Homo*, has just started the formation of its LRP4 and is at the stage of 3 unfused cusps (See Suppl. Fig. S11). Its age at death was histologically estimated to be 795 days, or 2.18 years [7].

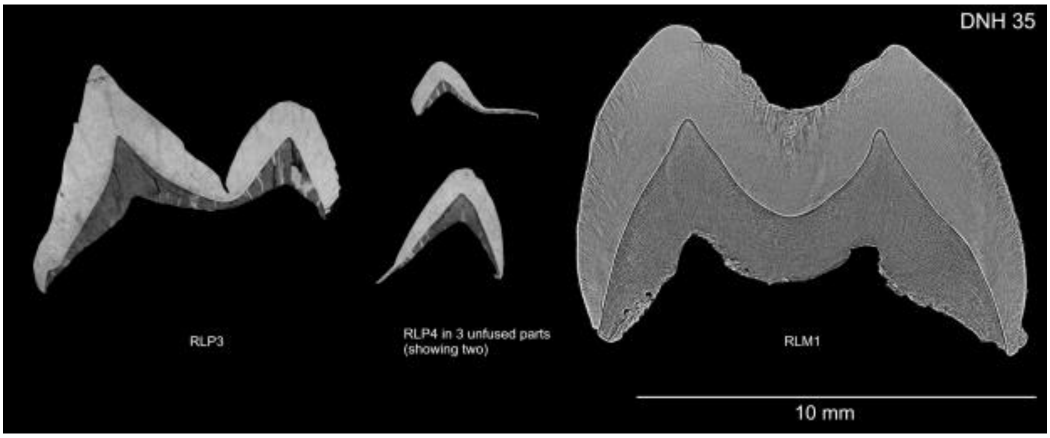

Supplementary Figure S11 –Virtual 2D section through the developmental plane the permanent tooth germs of South African specimen DNH 35 (after Fig. O in S1 file of Smith et al., 2015[7]).

Ramirez Rozzi[15,16] investigated the enamel microstructure of Plio-Pleistocene hominid teeth and concluded that premolars crowns form in 2.54 ± 0.35 years, while molars crowns form in 2.56 ±0.41 years. He further demonstrates that the ratio between the molar and the premolar crown formation times is ~1.01 in Plio-Pleistocene hominids, while it is at 0.77 in modern humans and ranges from 0.6 to 0.75 in great apes [15]. In StW 151, both P4 and M2 seem to reach crown completion at the same time (See Fig. Q in file S1 in [7]). In *Paranthropus robustus* DNH 108, both teeth also seem to have competed their crown at roughly the same time, at ~4.6 years for M^2 (See Fig. N in file S1 in [7]). In DNH 84 (*P. robustus*), the ULP4 has just fused its two cusps and likely completed its cuspal enamel by the time of death at 816 days or 2.2 years (See Fig. L in file S1 in [7]).

In the absence of any histological data for GAR IVE, one can assume an initiation of its LRP4 **shortly after 2 years of age.**

1.3.2. Linear measures stress to dentine horns

On Fig. S1, cuspal enamel thickness in the GAR IVE LRP4 reaches 1363 µm on the lingual cusp and 1 251µm on the buccal cusp. From Fig. Q in Smith et al. [7] (see Suppl. Fig. S12), cuspal thickness can be measured in StW 151: 2388 µm for the lingual cusp, and 2260 µm for the buccal cusp. GAR IVE would thus have just completed 55% of its cuspal enamel.

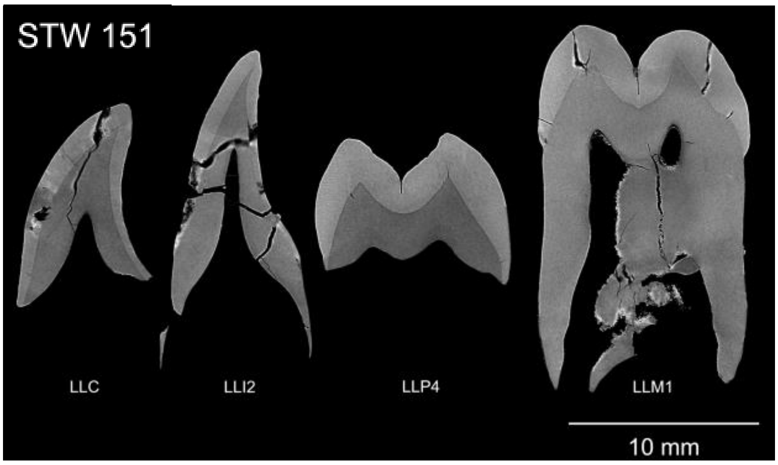

Supplementary Figure S12 –Virtual 2D section through the developmental plane the permanent tooth germs of South African specimen StW 151 (after Fig. Q in S1 file of Smith et al., 2015[7]).

Four accentuated lines (S1 to S4) were identified in the cuspal enamel of the LRP4. In spite of thorough attempts to optimize their visibility by tuning rotation in x,y,z and slice thickness, these accentuated lines could not be identified on a slice passing through the true dentine horn tip. Instead, the virtual thick slice passes through an EDJ ridge (Suppl. Fig. S13). The distance of these lines from the EDJ crest was measured. Keeping in mind the assumed achieved cuspal thickness observed in StW151, S1 to S3 would be in the inner cuspal enamel, while the remainder (including S4) would belong to the middle cuspal enamel.

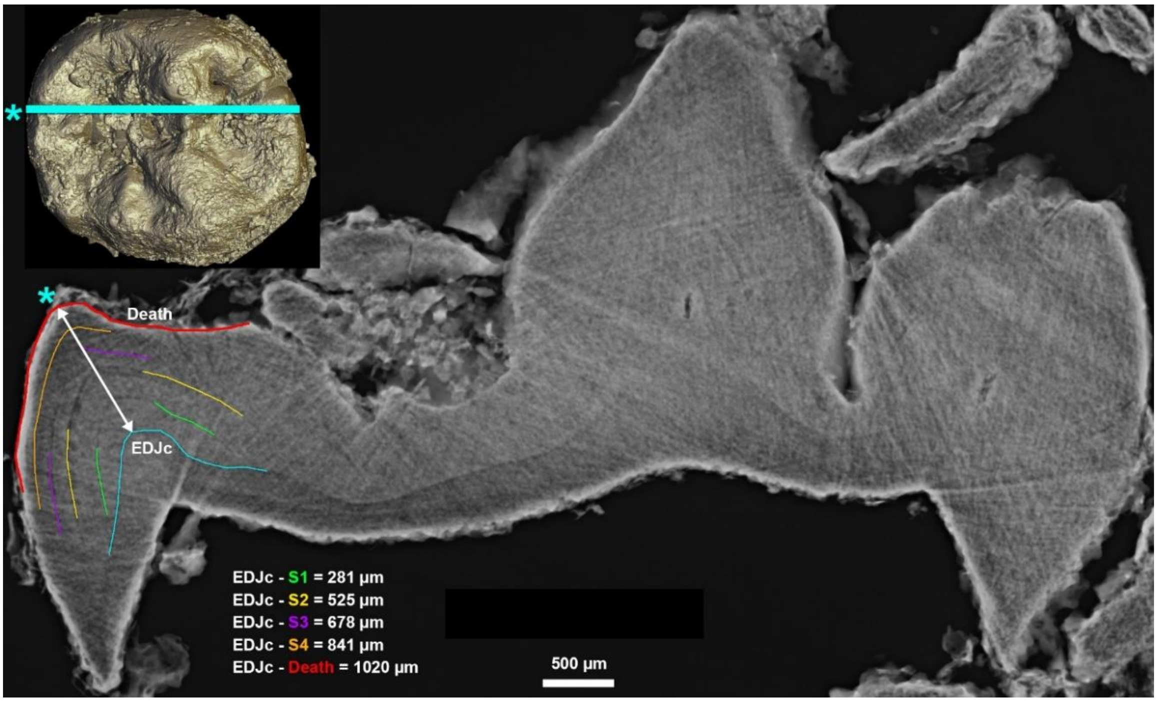

Supplementary Figure S13 – Virtual section (200 µm thick) though the lingual cusp of the GAR IVE LRP4. The orientation of the 2D slice is shown as a blue line on the 3D model. The cusp of interest is marked by an asterisk. Four accentuated lines (S1 to S4) were identified in the cuspal enamel, and their distance to the EDJ crest (EDJc) was measured.

1.3.3. Time of formation of the 4 accentuated lines

Abbreviations used to the following sections:

EDJc: EDJ crest.

S1, S2…, S4: Linear distance in µm between the EDJc and each stress event.

a) Published regression equation for enamel formation time in early *Homo*

Dean et al.[17] have published lowess regression equations for enamel formation rates in hominins. They especially report an equation based on six early *Homo* specimens:

$$y = 3.76 + 0.26x - 0.00002x^2, (R^2 = 0.99, s.e. = 0.01, P< 0.0001)$$

Based on the linear distances measured in 1.3.2., this equation yields the following times of formation:

Lingual cusp	Distance (µm)	Time (day)	Time (month)	Time between stress events (months)
**EDJc**	0			
**S1**	281	75.2	2.51	
**S2**	525	134.7	4.49	2.0
**S3**	678	170.8	5.69	1.2
**S4**	841	208.3	6.94	1.2
**Death**	1020	248.2	8.27	1.3

Supplementary Table S4 – Distances from the accentuated markings to the EDJ crest of the lingual cusp of GAR IVE LRP4, and corresponding formation time using Dean et al.[17]'s regression formula.

The stress events occur on average every ~1.4 months (Suppl. Table S4), whatever their cause (e.g., illness, food deficiencies).

b) Published enamel daily secretion rates (DSR) for early *Homo*

In their appendix 1, Lacruz et al. [18] published enamel daily secretion rates (DSR) for two African early *Homo* specimens. Because the stress in the GAR IVE LRP4 are located in the cuspal enamel, we consider only their cuspal DSR (Suppl. Table S5).

				**Cuspal DSR**				
Taxon	Tooth	Specimen	**Cu.out**	**Cu.mid**	**Cu.inn**		**Average per specimen**	**Overall average**
H. habilis	PM	KNM-ER 1805	5.21	4.6	3.68		**4.50**	
H. erectus	PM	KNM-ER 3733	5.28	4.54	4		**4.61**	**4.55**
**Average per anatomical region**			**5.25**	**4.57**	**3.84**			
		**Overall average**		**4.55**				

Supplementary Table S5 – Cuspal daily secretion rates collected from Lacruz et al.[18] (in µm/day), and average values.

Applying their average cuspal DSR rate of 4.55 µm/day to the linear distances of each stress to the EDJ crest tip yields the associated formation time (Suppl. Table S6).

**Lingual cusp**		mean Cu.DSR =	4.55	µm/d
	**Distance (µm)**	**Time (days)**		
**EDJc**	0			
**S1**	281	61.8		
**S2**	525	115.4		
**S3**	678	149.0		
**S4**	841	184.8		
**Death**	1020	224.2		

Supplementary Table S6 – Formation times of the accentuated marking identified in the GAR IVE LRP4 calculated using the average cuspal DSR (data from Lacruz et al.[18], see Suppl. Table S5).

Considering that S1 to S3 are in the inner cuspal enamel, and S4 to death in the middle cuspal enamel, one can apply the local rates provided by Lacruz et al.[18] (Suppl. Table S7).

**Lingual cusp**		**Distance (µm)**	**Time (days)**
Inner cuspal DSR = 3.84 µm/day	**EDJc**	0	
	**S1**	281	73.2
	**S2**	525	136.7
	**S3**	678	176.6
Middle cuspal DSR = 4.57 µm/d	**S4**	841	184.0
	**Death**	1020	223.2

Supplementary Table S7 – Formation times of the accentuated marking identified in the GAR IVE LRP4 calculated using local cuspal DSR from Lacruz et al.[18], and reported in Suppl. Table S5.

c) Cross-validation and comparison of the two approaches

The overall mean difference between formation times calculated using Dean et al.[17]'s equation and Lacruz et al.[18]'s average cuspal DSR remains acceptable, at ~20 days (Suppl. Table S8). The difference gets even smaller when using the local rates ~9 days, the best result being when using inner cuspal enamel DSR.

	Time of formation for the 4 stress events (in days)				
	Dean et al. (2001)	Lacruz et al. (2008) *average* CuDSR	Difference	Lacruz et al. (2008) *local* CuDSR	Difference
**EDJc**					
**S1**	75.2	61.8	13.5	73.2	2.1
**S2**	134.7	115.4	19.4	136.7	-2.0
**S3**	170.8	149.0	21.8	176.6	-5.7
**S4**	208.3	184.8	23.4	184.0	24.2
**Death**	248.2	224.2	24.0	223.2	25.0
			**20.4**		**8.7**
			(= average)		(= average)

Supplementary Table S8 – Cross-validation of the calculation for the formation times of the accentuated marking identified in the GAR IVE LRP4 by comparing values obtained using three different methods (see Suppl. Tables S4, S6, S7).

We choose to further use the times calculated using Dean et al. [17]'s equation.

1.3.4. Age of the five stress events

Assuming the initiation time of P4 (~2 years) and that the EDJ crest where the virtual slice was recorded formed ~3 months after the dentine horn tip, these were added to the *times* of formation of each stress line will yield an *age* of formation (Suppl. Table S9).

Assumed initiation of P4 in early *Homo:* **ca. 2 years + ~0.25 year (EDJ crest)**		
	Dean et al. (2001)	Age of formation of the stress events (years)
**EDJc**		2.2
**S1**	75.2	2.5
**S2**	134.7	2.6
**S3**	170.8	2.7
**S4**	208.3	2.8
**Death**	248.2	2.9

Supplementary Table S9 – Estimation of the age of formation of the accentuated marking observed in GAR IVE LRP4.

When superimposed with the lingual cusp of the GAR IVE LRP4, the most developed cusp of the DNH 35 LRP4 matches with the purple stress "S3" in GAR IVE (see **1.3.2.** and Suppl. Fig. S12), which, following the DNH 35 growth pattern, would have then occurred at ~the 796th day of life of GAR IVE (i.e., 2.18 years). Death occurred~77 days

later in GAR IVE, which correspond to ~873 days or ~2.4 years in the DNH 35 growth pattern. “S3” shows that the calculated estimation is slightly ahead of the developmental stage observed in other early *Homo* specimens (here by 6.5 months at most), yet this is may be accounted for by the variability which is not captured here, and by the uncertainties related to our measurements (e.g., the virtual section does not pass through the dentine horn tip).

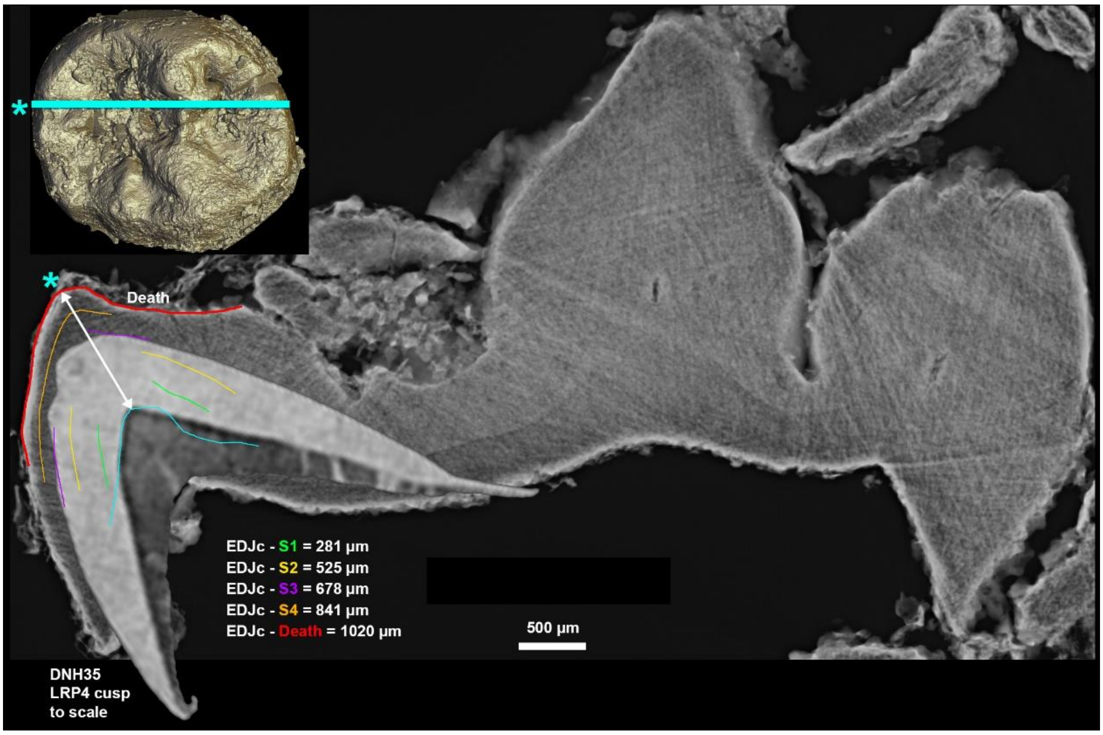

Supplementary Figure S14 – Most developed cusp of the DNH 35 LRP4 (Adapted from Figure O in Smith et al., 2015 [7]) to scale and superimposed to the GAR IVE lingual cusp. The time of death in DNH35 matches the accentuated line S3 in GAR IVE LRP4.

2. Taphonomic investigation of the early Homo *GAR IVE mandible and associated faunal remains. (Suppl. Text S2)*

Surface features

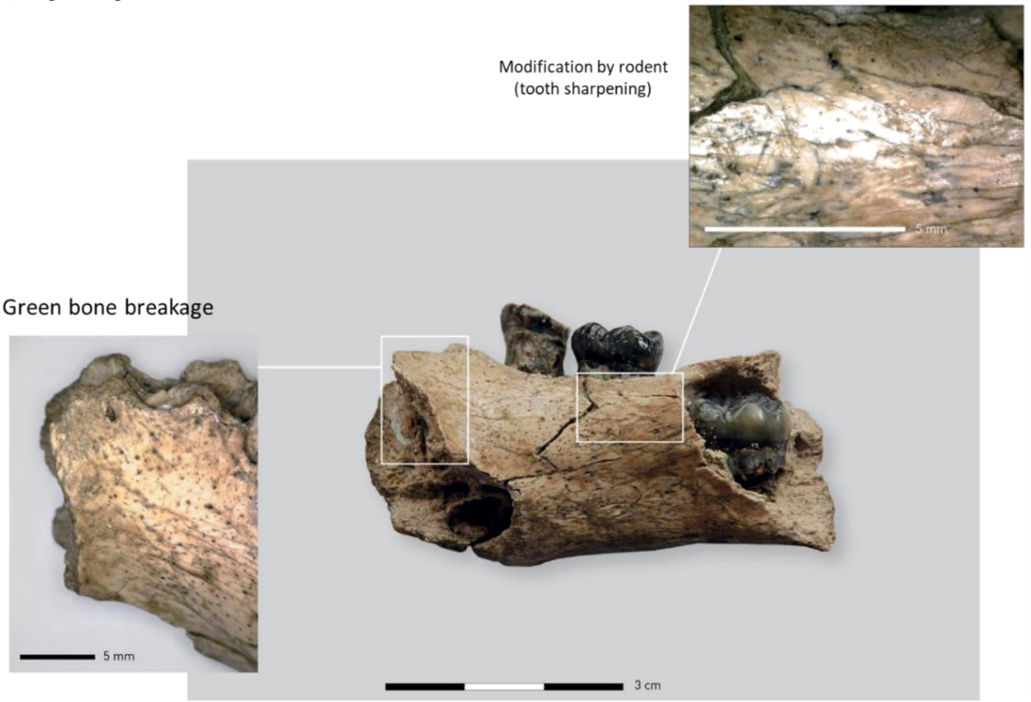

Supplementary Figure S15 – Bone surface modifications on the lingual aspect of the GAR IVE mandible.

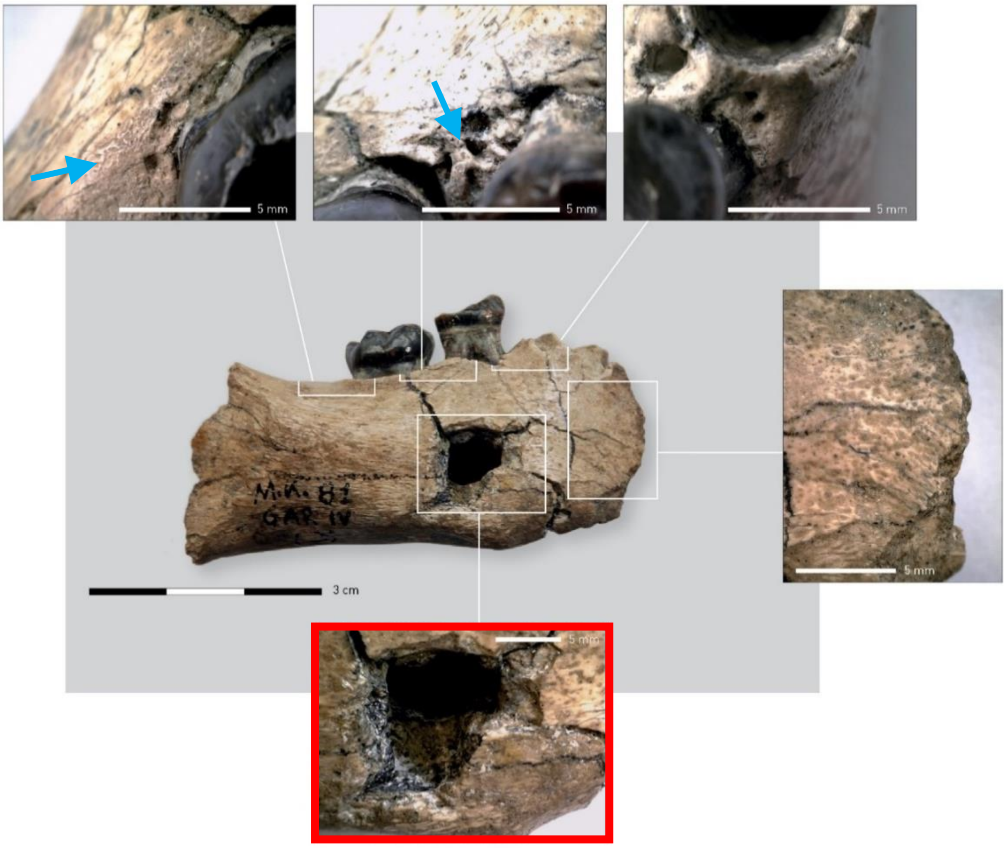

Supplementary Figure S16 – Bone surface modifications on the buccal aspect of the GAR IVE mandible showing the bone perforation in place of the mental foramen (red framed insert), slight weathering on the posterior aspect of the corpus. Note the damages (blue arrows, see Table S12) referred to as „Surface pit,

Class 1" by Parkinson[19], showing striations radiating from the outer circumference of the depression. See also Courtenay et al.[20].

Investigating the role of necrophagous insects in the taphonomic damages observed on GAR IVE.

Dermestids beetles marks on bones represents a particular point in the history of remains, and provides information on the taphonomic processes, including possible season of death[21]. Their occurrence noticeably indicates carcass exposure under warm conditions for several weeks to months and the presence of desiccated tissues during this period[21].

These insects produce various traces, like surface tunnels, pits, and bore holes[19,22]. Such traces have been reported on dinosaur, rhinoceros and *Stegomastodon* bones[22–26], *Bison latifronshorns*[21] and human bones from Bronze Age[27].

Larder beetles (Coleoptera: Dermestidae) are small (0.5 to 1 cm long) necrophagous beetles with a worldwide distribution. Hundreds of species have been reported worldwide, but less than 10 are currently observed on human remains[28]. They are mostly observed on dry corpses, particularly in indoor cases[29,30] or dry outdoor environment[28,31]. On the contrary, dermestid activity does not occur on submerged or buried carcasses[28]. Both larvae and adults are negatively phototrophic and most activity takes place in shaded places.

The larvae feed on dry necrotic tissues, yet bury themselves in a safe place before each molt. After 7 to 8 molts, they stop feeding and dig pupation chambers for nymphosis[32]. These chambers are excavated into adjacent compact surfaces including hard materials such as wood and bone[33]. Two kinds of dermestid traces on bones have been observed: osteophagy and burying of pupation chambers; both can occur at the same time and places[32].

Martin & West[21] defined the following criteria to diagnose dermestid pupation chambers:

(1) The creation of pupation chambers is always associated with presence of carrion.

(2) Pupation chambers on a single bone and necessarily of a given class are uniform in shape and fall within a certain size range (do not vary greatly in size).

(3) Pupation chambers should generally be found in closely associated groups.

(4) Because chambers are usually formed under desiccated tissue that later decays, they are usually found as half casts rather than completely enclosed burrows.

(5) Chambers are excavated by the mandibles and their surface tends to be covered with microscopic depressions representing mandible bites. These

are expressed as small bumps on the casts (these are best studied as casts of the original chamber).

(6) Pupation chambers usually are not much longer than the length of the pupa itself. They are flask shaped with the opening slightly constricted and the main chamber expanded slightly to fit the pupa.

(7) Dermestids, within a species, are fairly uniform in size and the girth of the chamber is a reliable clue for determining if traces found on bone are produced by dermestids.

A close examination of the GAR IVE early *Homo* mandible allows scoring the following features (Suppl. Table S10).

Criteria	FOR	AGAINST	Comments
1	Obvious		
2	Just one chamber, impossible to check		
3		Just one chamber found	Just a small part of the whole skeleton. Pupation chambers were found on faunal remains (see Supplementary Table S12).
4		This chamber looks complete.	Presence of a pre-existing vascular hole (mental foramen).
5		No visible microscopic depressions.	They may have been removed by taphonomic processes or cleaning.
6		The hole is quite deep, not bottle-shaped.	Presence of a pre-existing vascular hole (mental foramen).
7	Internal width of the chamber: 3.4-5 mm		Internal width of the chamber compliant with previous observations (Supplementary Fig. S17), but the outer width is larger than expected (8.02 mm). However, there was a pre-existing vascular hole (mental foramen).

Supplementary Table S10 – Analysis of the Garba IVE early *Homo* mandible according to the 7 criteria defined by Martin & West [21] to diagnose dermestid pupation chambers.

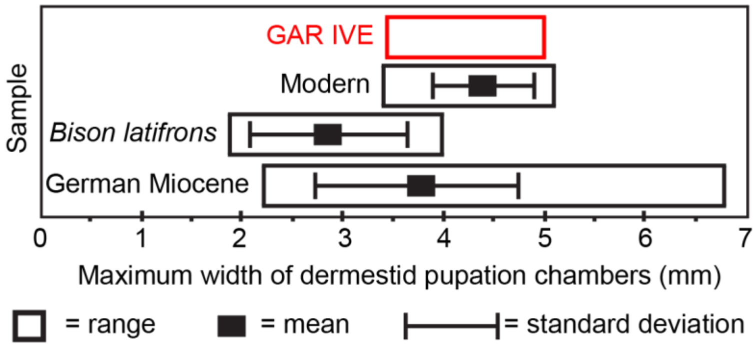

Supplementary Figure S17 – Maximum width of dermestid pupation chambers in modern and palaeontological material, in comparison with the observation made on GAR IVE (modified after Fig. 3 in [21]).

Parkinson[19] also stressed that only the co-occurrence of varying damage types allows for dermestids to be addressed as a variable for bone modification in a fossil faunal assemblage.

Accordingly, and to determine the agent at the origin of the bone perforation seen on the GAR IVE early *Homo* mandible (Figs. 2, 4), a taphonomic study of the associated faunal remains was undertaken. The small sample associated with the early *Homo* mandible originates from layer E and was uncovered during field seasons 2005 and 2009 as well as 1974 and 1975. It was analysed by Fiore and Tagliacozzo[34] (Suppl. Table S11) and is best described as a palimpsest.

However, based on very characteristic damages, 22 specimens can be attributed to the same time-slice within the chain of taphonomic events. Following the nomenclature outlined by Parkinson[19], one can observe surface tunnels (on 9 specimens), bore-holes (on 3 specimens) and surface pits (on 18 specimens). Three specimens showed all three damage-types represented (in red font in Suppl. Table S12).

To note that three specimens show gnawing damage by a small carnivore. Clear indication for bone modification by hominins is not evident.

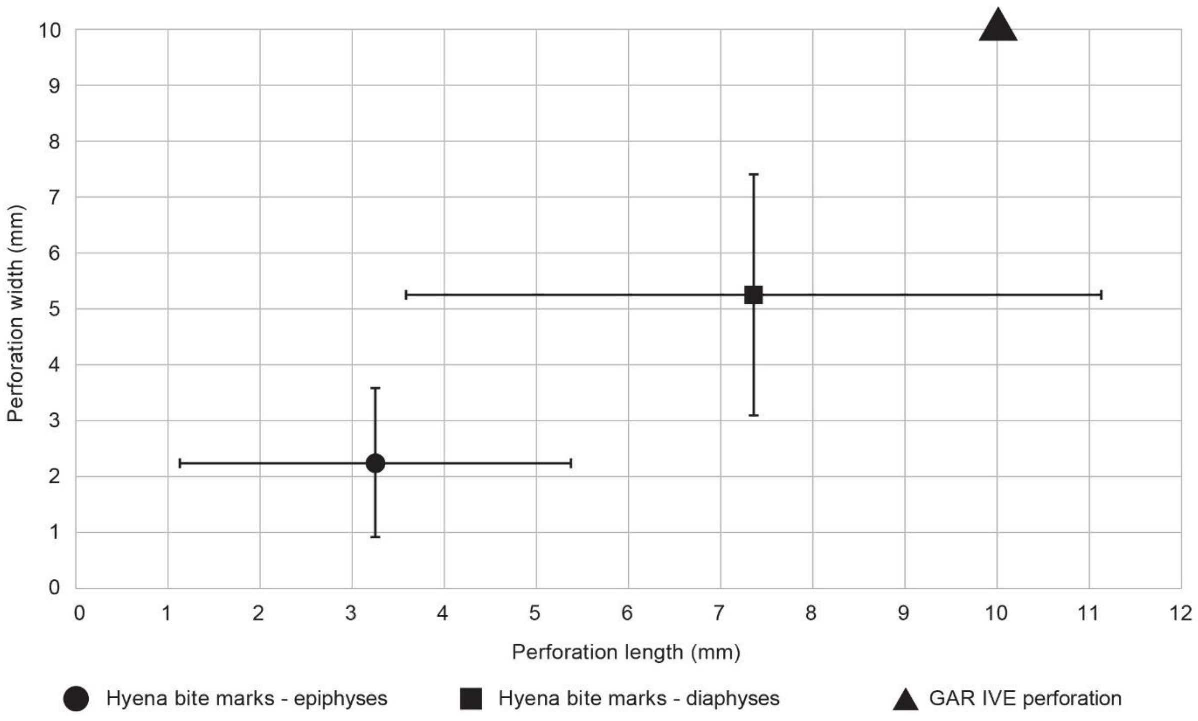

Supplementary Figure S18 – Plot of the size of the Garba IVE perforation on the buccal aspect of its mandibular corpus, in relation to the known dimensions of hyena bite marks on epiphyses and diaphysis as published by Dominguez-Rodrigo and Piqueras[35].

			Sample 2005/2009				Sample 1974/1975 (Fiore & Tagliacozzo 2004)			
**Tribe**	**Family/Order**	**Genus**	**SC**	**NISP**	**MNI**	**Total 2005/2009**	**NISP**	**MNI**	**Total 1974/75**	**Total sample (n=455)**
Carnivora	Mustelidae	*Enhydriodon aethiopicus*		0		0	1	1	1	**1**
Carnivora				1	1	1	0	0	0	**1**
Equidae	Equus	*Equus* sp.	3	8		8	0	0	0	**8**
Hippopotamidae	Hippopotamus L.	*Hippopotamus* sp.	5	28		28	7		7	**35**
		Hippopotamus cf. *amphibius*	0	0	0	0	1	1	1	**1**
Suidae							1		1	**1**
		Kolpocherus majus	3	1		1	1		1	**2**
Bovidae				11		11	2		2	**13**
	Reduncini	*Kobus* sp.	2	2		2	0	0	0	**2**
	Alcelaphini		4	15		15	1		1	**16**
		Connochaetes sp.	4	5		5				**5**
		Damaliscus sp.	3	0	0	0	1	1	1	**1**
	Antilopini	*Gazella* sp.	2	1		1				**1**
Aves				2	1	2	1		1	**3**
Total tax. det.						74			16	**90**
Indeterminate			1	3		3	2		2	
			2	35		35			0	
			3	23		23	12 *		12	
			4	13		13			0	
			5	11		11	9		9	
			indet.	221		221	7		7	
			not rec.	24		24	5		5	
Total tax. indet.						330			35	**365**

Supplementary Table S11 –Faunal sample composition for Garba IVE.

SC= Size Classes, NISP = Number of identified specimen per taxon, MNI = Minimum Number of Individuals.

*The number of 12 specimens represents large-medium sized (n=5), medium-sized (n=6) and medium-small sized (n=1) taxa [34].

No	Taxon / Size -Class	Fragment	Surface tunnel	Bore hole	Surface pit 1	Surface pit 2	Surface pit 3
05-22	indet. / 4	longbone				x	
05-31	indet. / 3	longbone				x	
05-32	indet. /3	long	x				
05-40	indet. / 3		x	x		x	
05-42	indet. / 2	longbone	x			x	
05-57	indet. / 2	vertebra	x	x			x
05-59	indet. / 3	vertebra					x
05-63	indet. / -	longbone				x	
05-66	indet. / -					x	
05-69	indet. / 3	longbone	x				
05-117	*Hippopotamus sp.* / 5	Mpp				x	
05-364	indet. / 3	longbone	x				x
05-383	*Hippopotamus sp.* / 5	molar				x	
09-1324	indet. / -	longbone	x				
09-1344	indet. / 2	tooth				x	
09-1378	indet. / -						x
09-1604	indet. / -					x	
09-1606	indet. / -	long	x	x		x	
09-1644	indet. / -	long	x				
09-1766	indet. / 3	skull				x	
09-1830	*Hippopotamus* sp. / 5	phalanx				x	

Supplementary Table S12 – Faunal remains with modifications defined by Parkington [19] as diagnostic for dermestid damage. Shaded rows highlight specimens with three types of modifications.

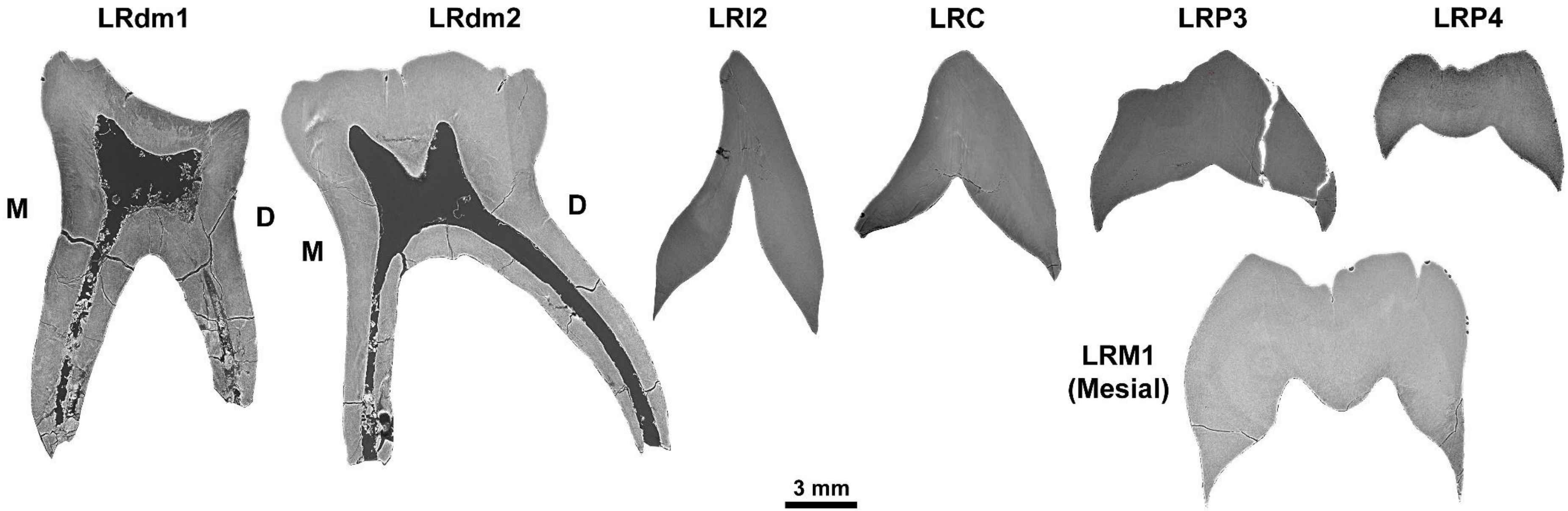

Supplementary Figure S19 –Virtual 2D sections through the GAR IVE teeth showing that there are no significant fractures that could have happened ante-mortem. The teeth are robust and do not look weakened by any pathology. Only a few post-mortem cracks can be seen in the roots of the deciduous molars and on the P3.

3. Reconstruction of the palaeoenvironment. (Suppl. Text S3)

Melka Kunture (8°42'N; 38°35'E) is located 60 km south of Addis Ababa, in a tectonic depression of the Upper Awash Valley of Ethiopia. This is a cluster of paleontological and archaeological sites within a volcanic area active all over the Early and Middle Pleistocene. During Quaternary times, the meandering paleo-Awash River transported and deposited sediments with variable grainsizes. Tephra generated by nearby volcanic eruptions accumulated in the area, clogging up the streams, contributing to the formation of shallow ponds and pools. Ephemeral streams currently erode the Pleistocene deposits and cut gullies, such as the Garba gully, where the site named Garba IV is located, exposing the stratigraphic sequence in natural incisions and allowing identification of archeological deposits which are the focus of excavations. Sedimentological facies suggest a fluvial setting where sedimentation was controlled by the reworking of volcanic material eroded upstream. Fluvial sedimentation of the paleo-Awash alternated with primary volcanic distal fallouts of ashes from the Early Pleistocene Melka series[36].

In Early Pleistocene times, the meandering upper paleo-Awash flowed through a gently undulating landscape[37]. The fluvial channel was slightly further south than the modern river course. The exposed stratigraphic sequence of the Garba IV site starts with silts and sands interbedded with rhyolitic volcanic ash layers and pumice lapilli providing evidence of low-energy fluvial sedimentation in an active volcanic area (Fig. 1c). Mottles suggest periodic backwater. Above, a deposit of pebbles within a sandy matrix (Layer E) is interpreted as the lag deposit of a fluctuating seasonal river. Abundant lithic implements and faunal remains were discovered within this layer. The hominin mandible, which laid on the margin of the river bank, is also part of the record (Fig. 1d). A thin sand deposit capped Layer E and was overlain, in turn, by a primary, partially eroded rhyolitic ash layer, dated at ~1.7 Ma[38].

The fluvial-volcanic context of the Melka area, with its high sedimentation rates provided essential conditions for fast burying of the GAR IVE remains. Acid rains and water could also have affected fossilization processes. In active volcanic regions, ash falls affect both the chemistry of rain and ground waters[39,40]. Waters contaminated by ash leaching are typically enriched in chloride, sulphate and fluoride anions[41]. In modern volcanic settings, ground waters can reach pH as low as 2[42]. In analogy to modern processes, when the late Oldowan layer containing the infant mandible was immersed in a humid environment, it was permeated by water with reduced pH due to leaching through the volcanic ash layers and contamination by volcanic aerosol derived from degassing and eruptions of the volcanoes at short distance from Melka Kunture[40,41].

4. Technical parameters for the μCT data acquisitions. (Suppl. Text S4)

4.1. PPC-SRμCT at the ESRF of GAR IVE.

The GAR IVE mandible was scanned using PPC-SR-μCT on the beamline ID 19 at the ESRF (Grenoble, France). First, an overview scan of the whole specimen was performed at ~25 μm, then the dentition was also scanned at ~6 μm (Suppl. Table S13).

	**Overview at ~25 μm**	**Overview at ~6 μm**
Date	11/12/2015	12/12/2015
Beamline	ID 19	ID 19
Operator	Dr. Paul Tafforeau	Dr. Paul Tafforeau
Storage ring operating mode (beam delivery)	16 bunch	16 bunch
Energy	100 keV	100 keV
Scintillator	LuAG 2000	LuAG 500
Filters	2.8 mm Al, 6.5 mm Cu	2.8 mm Al, 6.5 mm Cu
Wiggler	W150 gap at 66 mm	W150 gap at 63.2 mm
Phase distance	6400 mm	4400 mm
Synchrotron current	90 mA	70.73 mA
Camera	PCO Edge	PCO Edge
Optic	LAFIP 2	Hasselblad 6 μm
Pixel size	25.37 μm	6.34 μm
Number of projections	2499	8000
Scanning geometry	Normal (No half-acquisition)	Half-acquisition (1000 pixels offset)
Scanning mode	Continuous	Continuous
Number of scans	18	12
Time per scan	2 min 48s (including deadtime of 30s)	7.2 min
Total scanning time	1 h	1 h 40 min
Safety triggering time	1 ms	1 ms
Δz (height per scan)	3.4 mm	5 mm
Exposure time	0.05 s	0.05 s
Darks (no beam)	400 (only once, at the beginning of the acquisition)	100 (at the end)
References (sample out of the beam)	401 (only once, at the beginning of the acquisition)	101 (at the beginning and at the end).
Binning during the acquisition	None	None

Comments	- no contrast at all between dentine and enamel.<br>- ~Homogeneous grey level between bone, enamel and dentine, on both deciduous teeth and permanent tooth germs.<br>- density of enamel ≈ 2.90<br>- Hypothesis: bone and dentine have been remineralized (taphonomic remodeling). → Average density in LRC and LRI2 = 3, seems to confirm remineralization.	- still no contrast between enamel and dentine.<br>- EDJ is barely visible from place to place.<br>- No Retzius lines visible.<br>- Perikymata visible in very small areas on few tooth surfaces because of recristallisation occurring on the crowns (i.e., cristals will obliterate the perikymata).

Supplementary Table S13 – Technical parameters for the PPC-SR-µCT acquisition of the overview scans of GAR IVE on the ID 19 beamline at the ESRF.

Each permanent tooth was scanned at ~3.4 µm to image the incremental growth lines (Retzius lines). Last, high-resolution scans were acquired at 0.6 µm in the LRP4 and the LRC, to image the enamel microstructure (Suppl. Table S14).

	Tooth scan at 3.4 µm	High-resolution for enamel microstructure in the LRC and LRP4
Date	13/12/2015	13/12/2015
Beamline	ID 19	ID 19
Operator	Dr. Paul Tafforeau	Dr. Paul Tafforeau
Storage ring operating mode (beam delivery)	16 bunch	16 bunch
Energy	100 keV	~50 keV
Scintillator	LuAG 200	GGG 10
Filters	2.8 mm Al, 5 mm Cu	5.6 mm Al, 0.7 mm Cu, 1 mm diamond
Wiggler	Wiggler W150 gap at 58 mm	Insertion device gap: U176c = 159.9937; W150BT = -0.0314 ; W150B = 259.9931; PPU32A = 11.1012
Phase distance	3400 mm	200 mm
Synchrotron current	77.86 mA	79.9 mA
Camera	PCO Edge	PCO EDGE CAMERALINK (SN 1468)

Optic	Rodenstock	OP-x10-x1 with a lead glass meniscus to protect the optic.
Pixel size	3.41 µm	0.637 µm
Number of projections	8000	8000
Scanning geometry	Half-acquisition (1000 pixels offset)	Half-acquisition (1000 pixels offset)
Scanning mode	Continuous	Continuous
Number of scans	13	1
Time per scan	-	-
Total scanning time	-	-
Safety triggering time	1 ms	5 ms
Δz (height per scan)	5 mm	-
Exposure time	0.1 s	0.1 s
Darks (no beam)	100 (at the end)	100 (at the end)
References (sample out of the beam)	101 (at the beginning and at the end).	101 (at the beginning and at the end).
Binning during the acquisition	None	None
Comments	A few of accentuated lines are visible from place to place mostly in the LRP4, and to a much lesser extent in LRP3 and LRC.	No visible microstructure.

Supplementary Table S14 – Technical parameters for the PPC-SR-µCT acquisition of the middle and high resolution scans of GAR IVE on the ID 19 beamline at the ESRF.

Volumes were reconstructed using a filtered back-projection algorithm (PyHST2 software, ESRF), and Paganin's approach ([43], see [44] for an application at the ESRF). Original 32-bit stacks were converted into 16-bit tiff stacks. The subscans were then concatenated, ring artifact correction was applied as required, and the concatenated subscans were cropped to define the final size (bounding box) of the dataset. Data were saved as JPEG 2000 with a compression factor of 10 (this format minimizes the loss of data quality).

4.2. Conventional µCT at MPI-EVA of the modern comparative samples (Suppl. Table S15).

	AI_1 (ULM3)	AI_2	AI_3
**Form of AI**	rather hypoplastic	?	Maybe hypocalcified
**Tooth type**	UM3	dm	ULdm2
**µCT scanner**	diondo d3	diondo d3	diondo d3

**Voltage (kV)**	120	130	130
**Current (µA)**	50	40	40
**Filters thickness (mm)**	0.5 mm Brass	0.5 mm Brass	0.5 mm Brass
**Integration time (ms)**	1500	1500	1500
**Number of projections**	2970	2970	2970
**Frame average**	2	2	2
**Pixel size (µm)**	5.8618	5.4820	5.4820
**Resampled pixel size for segmentation and 3D model (µm)**	15	15	15
**Scan duration**	14h 51min	2h30min	2h30min

Supplementary Table S15 – Technical parameters for the conventional µCT scan acquisitions of the modern human clinical teeth, in the Department of Human Evolution (MPI-EVA, Leipzig, Germany).

Volumes were reconstructed using a filtered back-projection algorithm, beam-hardening and ring artifacts correction were applied as needed. Data were saved as a stack of tiff files. The scans were resampled at 15 µm and filtered using a median and a Kuwahara filter (kernel size of 3 for each; [45]) to facilitate the segmentation in Avizo 6.3.1 (VSG). Enamel thickness was visualized in 3D by computing distances points by point between the enamel and dentine surfaces.

5. Cited references

1. Smith, T. M. *et al.* Dental evidence for ontogenetic differences between modern humans and Neanderthals. *Proc. Natl. Acad. Sci.* **107**, 20923–20928 (2010).
2. Condemi, S. The Garba IV E mandible. in *Studies on the Early Paleolithic site of Melka Kunture, Ethiopia* (eds. Chavaillon, J. & Piperno, M.) 687–701 (2004).
3. Zanolli, C. *et al.* Structural organization and tooth development in a *Homo* aff. *erectus* juvenile mandible from the Early Pleistocene site of Garba IV at Melka Kunture, Ethiopian highlands. *Am. J. Phys. Anthropol.* **162**, 533–549 (2017).
4. AlQahtani, S. J., Hector, M. P. & Liversidge, H. M. Brief communication: the London atlas of human tooth development and eruption. *Am. J. Phys. Anthropol.* **142**, 481–490 (2010).
5. Kuykendall, K. L. Dental development in chimpanzees (*Pan troglodytes*): The timing of tooth calcification stages. *Am. J. Phys. Anthropol.* **99**, 135–157 (1996).
6. Demirjian, A., Goldstein, H. & Tanner, J. M. A new system of dental age assessment. *Hum. Biol.* **45**, 211–227 (1973).
7. Smith, T. M. *et al.* Dental Ontogeny in Pliocene and Early Pleistocene Hominins. *PLoS ONE* **10**, e0118118 (2015).
8. Gunz, P. *et al.* *Australopithecus afarensis* endocasts suggest ape-like brain organization and prolonged brain growth. *Sci. Adv.* **6**, eaaz4729 (2020).

9. Dean, M. C. The dental developmental status of six East African juvenile fossil hominids. *J. Hum. Evol.* **16**, 197–213 (1987).
10. Leakey, R. E. F. Further Evidence of Lower Pleistocene Hominids from East Rudolf, North Kenya, 1972. *Nature* **242**, 170–173 (1973).
11. Bromage, T. G. & Dean, M. C. Re-evaluation of the age at death of immature fossil hominids. *Nature* **317**, 525–527 (1985).
12. Leakey, R. E. F. & Wood, B. A. New evidence of the genus *Homo* from East Rudolf, Kenya (IV). *Am. J. Phys. Anthropol.* **41**, 237–243 (1974).
13. Dean, M. C. & Smith, B. H. Growth and development of the Nariokotome youth, KNM-WT 15000. in *The First Humans: Origin and Early Evolution of the Genus* Homo (eds. Grine, F. E., Fleagle, J. G. & Leakey, R. E.) 101–120 (2009).
14. Dean, M. C. Growth of teeth and development of the dentition in *Paranthropus*. in *Evolutionary History of the "Robust" Australopithecines* (ed. Grine, F. E.) 43–53 (Aldine de Gruyter Publishing Co., 1988).
15. Ramirez Rozzi, F. V. Enamel growth markers in hominid dentition. *Eur. Microsc. Anal.* 21–23 (1994).
16. Ramirez Rozzi, F. Time of crown formation in Plio-Pleistocene hominid teeth. in *Aspects of Dental Biology: Palaeontology, Anthropology and Evolution* (ed. Moggi-Cecchi, J.) 217–238 (International Institute for the Study of Man, 1995).
17. Dean, C. *et al.* Growth processes in teeth distinguish modern humans from *Homo erectus* and earlier hominins. *Nature* **414**, 628–631 (2001).
18. Lacruz, R. S., Dean, M. C., Ramirez-Rozzi, F. & Bromage, T. G. Megadontia, striae periodicity and patterns of enamel secretion in Plio-Pleistocene fossil hominins. *J. Anat.* **213**, 148–158 (2008).
19. Parkinson, A. H. *Dermestes maculatus* and *Periplaneta americana*: bone modification criteria and establishing their potential as climatic indicators. (Faculty of Science, University of the Witwatersrand, 2012).
20. Courtenay, L. A. *et al.* Obtaining new resolutions in carnivore tooth pit morphological analyses: A methodological update for digital taphonomy. *PLOS ONE* **15**, 1–30 (2020).
21. Martin, L. D. & West, D. L. The recognition and use of dermestid (Insecta, Coleoptera) pupation chambers in paleoecology. *Palaeogeogr. Palaeoclimatol. Palaeoecol.* **113**, 303–310 (1995).
22. Britt, B. B., Scheetz, R. D. & Dangerfield, A. A Suite of Dermestid Beetle Traces on Dinosaur Bone from the Upper Jurassic Morrison Formation, Wyoming, USA. *Ichnos* **15**, 59–71 (2008).
23. Laudet, F. & Antoine, P.-O. Des chambres de pupation de Dermestidae (Insecta : Coleoptera) sur un os de mammifère tertiaire (phosphorites du Quercy) : implications taphonomiques et paléoenvironnementales. *Geobios* **37**, 376–381 (2004).
24. Roberts, E. M., Rogers, R. R. & Foreman, B. Z. Continental insect borings in dinosaur bone: examples from the Late Cretaceous of Madagascar and Utah. *J. Paleontol.* **81**, 201–208 (2007).
25. Bader, K. S., Hasiotis, S. T. & Martin, L. D. Application of forensic science techniques to trace fossils on dinosaur bones from a quarry in the Upper Jurassic Morrison Formation, northeastern Wyoming. *Palaios* **24**, 140–158 (2009).
26. Saneyoshi, M., Watabe, M., Suzuki, S. & Tsogtbaatar, K. Trace fossils on dinosaur bones from Upper Cretaceous eolian deposits in Mongolia: Taphonomic interpretation of

paleoecosystems in ancient desert environments. *Palaeogeogr. Palaeoclimatol. Palaeoecol.* **311**, 38–47 (2011).

27. Huchet, J.-B. *et al.* Identification of dermestid pupal chambers on Southern Levant human bones: inference for reconstruction of Middle Bronze Age mortuary practices. *J. Archaeol. Sci.* **40**, 3793–3803 (2013).
28. Charabidze, D., Colard, T., Vincent, B., Pasquerault, T. & Hedouin, V. Involvement of larder beetles (Coleoptera: Dermestidae) on human cadavers: a review of 81 forensic cases. *Int. J. Legal Med.* **128**, 1021–1030 (2014).
29. Voigt, J. Specific post-mortem changes produced by larder beetles. *J. Forensic Med.* **12**, 76–80 (1965).
30. Schroeder, H., Klotzbach, H., Oesterhelweg, L. & Püschel, K. Larder beetles (Coleoptera, Dermestidae) as an accelerating factor for decomposition of a human corpse. *Forensic Sci. Int.* **127**, 231–236 (2002).
31. Lefebvre, F. & Gaudry, E. Forensic entomology: a new hypothesis for the chronological succession pattern of necrophagous insect on human corpses. *Ann. Société Entomol. Fr. NS* **45**, 377–392 (2009).
32. Sommer, H. G. Cleaning skeletons with dermestid beetles—two refinements in the method. *Curator* **17**, 290–298 (1974).
33. Hinton, H. E. *A Monograph of the Beetles associated with stored Products. Volume I.* vol. I (London, Brit. Mus.(Nat. Hist.), 1945).
34. Fiore, I. & Tagliacozzo, A. Taphonomic analysis of the bone remains from the Oldowan site of Garba IV. in *Studies on the Early Paleolithic site of Melka Kunture, Ethiopia.* (eds. Chavaillon, J. & Piperno, M.) 639–682 (2004).
35. Domínguez-Rodrigo, M. & Piqueras, A. The use of tooth pits to identify carnivore taxa in tooth-marked archaeofaunas and their relevance to reconstruct hominid carcass processing behaviours. *J. Archaeol. Sci.* **30**, 1385–1391 (2003).
36. Kieffer, G., Raynal, J.-P. & Bardin, G. Cadre structural et volcanologique des sites du Paléolithique ancien de Melka Kunture (Awash, Ethiopie): premiers résultats. in *Hommes et Volcans. De l'éruption à l'objet* (eds. Raynal, J.-P., Albore-Livadie, C. & Piperno, M.) 77–92 (2002).
37. Raynal, J.-P. & Kieffer, G. Lithology, dynamism and volcanic successions at Melka Kunture (Upper Awash, Ethiopia). in *Studies on the Early Paleolithic site of Melka Kunture, Ethiopia* (eds. Chavaillon, J. & Piperno, M.) 111–135 (2004).
38. Morgan, L. E. *et al.* A chronological framework for a long and persistent archaeological record: Melka Kunture, Ethiopia. *J. Hum. Evol.* **62**, 104–115 (2012).
39. Harding, D. & Miller, J. M. The influence on rain chemistry of the Hawaiian Volcano Kilauea. *J. Geophys. Res. Oceans* **87**, 1225–1230 (1982).
40. Robock, A. Chapter 53 - Climatic Impacts of Volcanic Eruptions. in *The Encyclopedia of Volcanoes* (ed. Sigurdsson, H.) 935–942 (Academic Press, 2015). doi:10.1016/B978-0-12-385938-9.00053-5.
41. Stewart, C. *et al.* Contamination of water supplies by volcanic ashfall: A literature review and simple impact modelling. *J. Volcanol. Geotherm. Res.* **158**, 296–306 (2006).

42. Cuoco, E. *et al.* Impact of volcanic plume emissions on rain water chemistry during the January 2010 Nyamuragira eruptive event: Implications for essential potable water resources. *J. Hazard. Mater.* **244–245**, 570–581 (2013).
43. Paganin, D., Mayo, S. C., Gureyev, T. E., Miller, P. R. & Wilkins, S. W. Simultaneous phase and amplitude extraction from a single defocused image of a homogeneous object. *J. Microsc.* **206**, 33–40 (2002).
44. Sanchez, S., Ahlberg, P. E., Trinajstic, K. M., Mirone, A. & Tafforeau, P. Three-dimensional synchrotron virtual paleohistology: a new insight into the world of fossil bone microstructures. *Microsc. Microanal.* **18**, 1095–1105 (2012).
45. Wollny, G. *et al.* MIA - A free and open source software for gray scale medical image analysis. *Source Code Biol. Med.* **8**, 20 (2013).
